# Nonspecific Binding of Adenosine Tripolyphosphate and Tripolyphosphate Modulates the Phase Behavior of Lysozyme

**DOI:** 10.1021/jacs.2c09615

**Published:** 2023-01-06

**Authors:** Matja Zalar, Jordan Bye, Robin Curtis

**Affiliations:** Manchester Institute of Biotechnology, Department of Chemical Engineering, Faculty of Science and Engineering, The University of Manchester, 131 Princess Street, ManchesterM1 7DN, U.K.

## Abstract

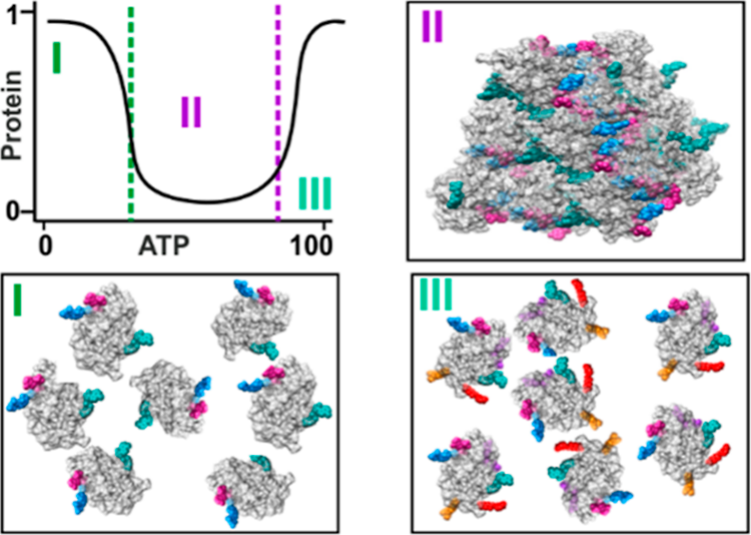

Adenosine tripolyphosphate (ATP) is a small polyvalent anion that has recently been shown to interact with proteins and have a major impact on assembly processes involved in biomolecular condensate formation and protein aggregation. However, the nature of non-specific protein–ATP interactions and their effects on protein solubility are largely unknown. Here, the binding of ATP to the globular model protein is characterized in detail using X-ray crystallography and nuclear magnetic resonance (NMR). Using NMR, we identified six ATP binding sites on the lysozyme surface, with one known high-affinity nucleic acid binding site and five non-specific previously unknown sites with millimolar affinities that also bind tripolyphosphate (TPP). ATP binding occurs primarily through the polyphosphate moiety, which was confirmed by the X-ray structure of the lysozyme–ATP complex. Importantly, ATP binds preferentially to arginine over lysine in non-specific binding sites. ATP and TPP have similar effects on solution-phase protein–protein interactions. At low salt concentrations, ion binding to lysozyme causes precipitation, while at higher salt concentrations, redissolution occurs. The addition of an equimolar concentration of magnesium to ATP does not alter ATP binding affinities but prevents lysozyme precipitation. These findings have important implications for both protein crystallization and cell biology. Crystallization occurs readily in ATP solutions outside the well-established crystallization window. In the context of cell biology, the findings suggest that ATP binds non-specifically to folded proteins in physiological conditions. Based on the nature of the binding sites identified by NMR, we propose several mechanisms for how ATP binding can prevent the aggregation of natively folded proteins.

## Introduction

Adenosine tripolyphosphate (ATP) is a small polyvalent anion with various roles in cell biology. It consists of a hydrophobic adenosine and a negatively charged tripolyphosphate (TPP) moiety. ATP is a primary energy source driving biochemical reactions in cells and acts as a signaling molecule in both extra- and intra-cellular signaling cascades that have been described in detail elsewhere.^1−7^ All living cells maintain 1–10 mM concentration of ATP at all times,^8,9^ which is much higher than that required to maintain the biochemical processes. Recently, it has been hypothesized that ATP is not only involved in the formation of membranelles organelles through liquid–liquid phase separation (LLPS) that allows control over complex biochemical reactions in time and space^10,11^ but also plays a role in the development of certain diseases, including cancer, neurodegenerative diseases, and even viral infections,^12,13^ by modulating LLPS behavior or inhibiting fibrillation of proteins that are causing these diseases.^14−16^ Additionally, ATP has been shown to suppress protein aggregation and amyloid formation in crowded environments^17−19^ and improve protein stability and solubility proteome-wide.^20^

The dramatic effects of ATP on protein self-assembly processes arise due to binding interactions that occur at millimolar concentrations of ATP. ATP is known to bind (with millimolar affinity) to RNA recognition motifs in RNA binding proteins and to RGG low-complexity domains (LCDs) in intrinsically disordered proteins.^17,21−24^ The LCDs are small stretches of unstructured amino acids containing a high content of glycine and basic residues, which interact non-specifically with nucleic acids. However, ATP interactions extend beyond nucleic acid binding sites on proteins. A proteome-wide study found that about 25 percent of the insoluble proteome of human Jurkat cells is solubilized by ATP.^20^ Only a small fraction of the solubilized proteins are known ATP binders, while most contain intrinsically disordered (IDR) regions rich in basic amino acids. Along these lines, studies on the LLPS behavior of TDP-43 have revealed that ATP interacts non-specifically with Arg-containing IDR regions on proteins, although interactions with lysine occur at a much lower affinity.^25^ The preference of the adenosine group for arginine over lysine has also been deduced by Alshareedah et al. who showed that Arg-enriched LCDs versus Lys-enriched LCDs exhibit a broader condensation regime in solutions with poly(A) but the same phase behavior in solutions with polyphosphate.^26^ The increased affinity of adenosine for arginine has been attributed to its capability to form cation−π and π–π interactions with arginine but only cation−π interactions with lysine. The results can in part explain why ATP, but not TPP, is effective in solubilizing the nuclear protein FUS,^18^ or in possibly preventing fibrillation of the amyloid beta protein fragment (Aβ42).^27^ However, there is other evidence to suggest that the difference between ATP and TPP cannot be rationalized only in terms of preferential adenosine–arginine interactions. TPP and ATP have similar effectiveness in preventing thermal-induced aggregation of arginine-containing proteins such as serum albumin and ovalbumin.^27,28^ TPP is also capable of forming preferential interactions with arginines over lysines in disordered protein regions.^29^ This was shown through a comparative phase behavior study as a function of TPP concentration for the IDP histatin-5 and a series of arginine–lysine mutants, where the width of the condensation region directly correlates with the arginine to lysine content of the mutant irrespective of where the mutations occur.

Molecular insight into the nature of the ATP binding sites has been gained through NMR and molecular simulations. Molecular simulation studies have shown that a key factor for ATP to suppress the fibrillation of Aβ42 is preferential interactions of adenosine with aromatic groups, such as tryptophan, stabilized through π–π stacking interactions,^27,30^ which could explain why TPP is ineffective as an aggregation suppressor. On the other hand, while ATP, but not TPP, is effective in resolubilizing the FUS protein, using ^15^N heteronuclear single quantum coherence (HSQC)-NMR, direct interactions of ATP were detected with the folded RNA recognition motifs as well as the RGG-rich LCDs but not the aromatically rich prion-like domain.^22,23^ The binding to an arginine-enriched IDR region of TDP-43 has also been detected through HSQC-NMR, but no direct binding has been observed for the arginine-rich globular eye lens protein γS-crystallin,^31^ which suggests that flexibility or disorder is a key factor in determining the binding propensity. That is not to say, binding does not occur in natively folded proteins. Nishizawa et al.^32^ detected multiple ATP binding sites by NMR on ubiquitin and ubiquitin domain-associated receptor p62 (UBA) in regions on the protein that are flexible and enriched in basic and hydrophobic residues. In particular, binding occurs with the c-terminal tails for both proteins and the loop region of ubiquitin. Similar deductions about binding site composition have been reached in a molecular simulation study of ATP interacting with ubiquitin, lysozyme, or malate dehydrogenase.^33^ While the study found that flexible-loop regions are most susceptible to ATP binding, more than 40% of the protein surfaces are covered by ATP molecules, suggesting more extensive binding than previously observed. A key finding from these studies is that many of the ATP binding sites do not have any biological function. These non-biological binding sites are referred to here as non-specific, even though the binding could still occur through the formation of a specific complex that is reminiscent of biological binding sites.

Understanding where and how the ions bind to the protein is critical toward understanding the impact on protein self-assembly. Studies predominantly focused on IDR-containing proteins have hypothesized that when IDR regions bind the adenosine group, the TPP moiety provides a protective layer of hydration and prevents protein association, which can be especially strong for aromatics or arginine groups due to their ability to form cation−π and π–π interactions.^18^ For natively folded proteins, molecular simulations indicate that binding to loop regions leads to a reduction in their flexibility. Because these groups are often the most susceptible to unfolding upon heating, it has been hypothesized that ATP binding not only increases the protein’s thermal stability but the unfolded regions are less likely to associate due to preferential interactions with ATP clusters.^32,33^ On the other hand, we showed that ATP or TPP binds to negatively charged proteins, leading to an overcharging effect and an increase in colloidal stability, which slows down aggregate growth rates.^28^ In contrast, for positively charged proteins like lysozyme and the IDP histatin-5, low concentrations of TPP have the opposite effect of causing protein precipitation.^29,34^ Similarly, ATP has been shown to increase fibril formation for a series of basic IDPs,^35^ as well as an insulin fragment conjugated to octalysine.^36^ In each of these cases, the ion-specific effects were attributed to polyphosphate forming ionic bridges between basic protein groups. As such, it is not clear what factors determine whether bound ATP molecules prefer to form clusters with other ATP molecules, thereby stabilizing the protein against self-association, or forming ion bridges to a binding site on another protein molecule.

Future developments in cell biology require determining the extent of non-specific ATP binding to protein surfaces and their molecular basis. Nishizawa et al.^32^ used NMR to identify non-specific ATP binding sites on globular proteins, but the chemical shift data were not definitive enough to estimate binding constants. Furthermore, NMR does not directly capture the intermolecular interactions occurring between a ligand and a binding site. In this study, to clarify the molecular interactions involved in ATP binding to proteins, we combined nuclear magnetic resonance (NMR) measurements of lysozyme in solutions containing either ATP, TPP, or adenosine with X-ray crystallography of lysozyme–ATP complexes. The high-resolution crystal structure indicates that there are three ATP binding sites on lysozyme. Using solution NMR, we identified six binding sites, including the three seen in the crystal, and determined there is one high-affinity site with a sub-millimolar dissociation constant (*K*_d_) and five medium-affinity sites with *K*_d_ ∼ mM. The crystal structure analysis combined with NMR studies using TPP and adenosine indicated that the majority of binding interactions occur through the triphosphate moieties, with a notable exception that the adenosine group contributes to the stabilization of the high-affinity site. To probe the impact of ion binding on inter-protein interactions, we have measured an apparent diffusion interaction parameter *k*_D_ and protein precipitation boundaries. We show that the presence of Mg^2+^ or Na^+^ ions at biological concentrations does not alter the interaction of ATP with lysozyme at individual binding sites but dramatically alters the assembly behavior of ATP–protein complexes.

## Results

### Re-entrant Condensation of Lysozyme Is Induced by TPP and ATP

Mapping of protein phase separation boundaries helps to identify specific conditions which may exhibit interesting phenomena. Previously, we have reported that TPP triggers reentrant condensation of lysozyme at high pH (pH 9.0),^34^ and here we extend this work by additionally studying effects of ATP at pH 7.0.

**Figure 1:**
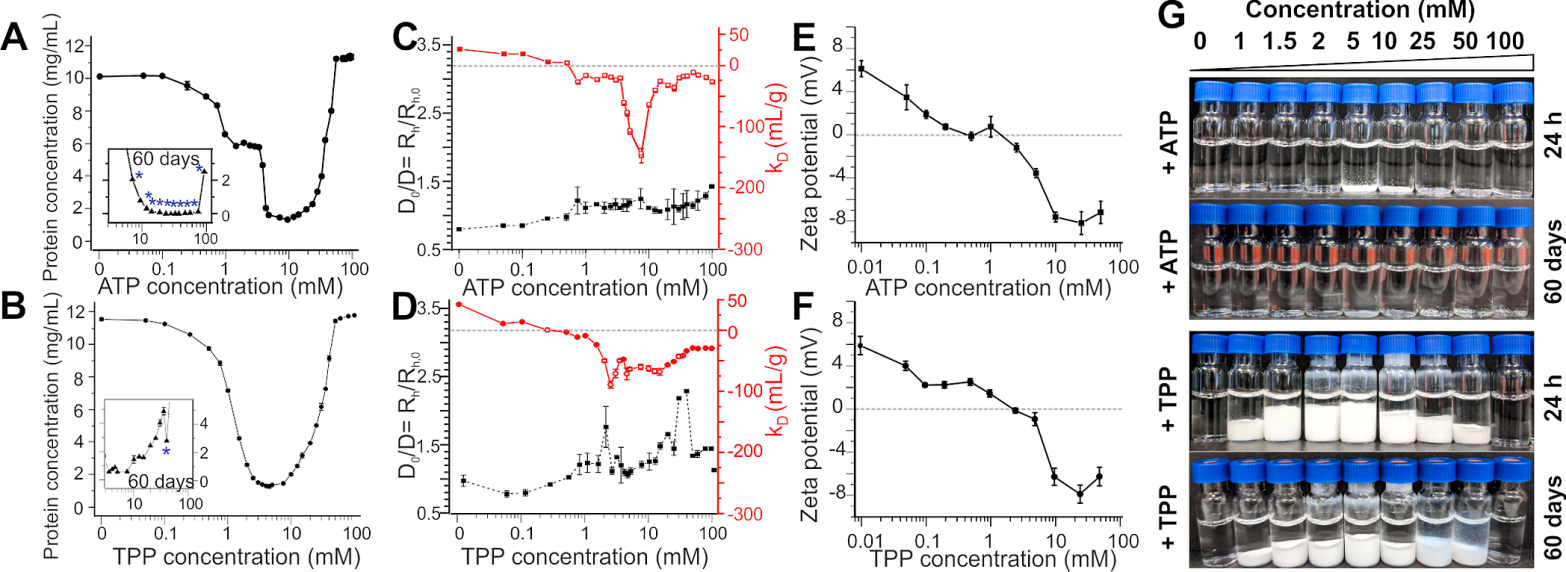
Biophysical characterization of lysozyme phase transition upon addition of ATP and TPP. (A,B) Protein concentration measurements immediately after sample preparation (black circles) of lysozyme solution upon addition of various concentrations of (A) ATP and (B) TPP. Figure inserts show protein concentration measurements (black triangles) in the ATP/TPP concentration region where crystal formation was observed after 60 days of incubation at room temperature (denoted with blue stars). (C,D) Inverse of the mutual diffusion coefficient normalized by the infinite dilution value *D*_0_/*D* or equivalently *R*_h_/*R*_h,0_ and estimate of the interaction parameter (*k*_D_) (red) measured by DLS of the soluble protein fraction after addition of various concentrations of (C) ATP and (D) TPP. (E,F) ζ-Potentials for lysozyme at various concentrations of (E) ATP and (F) TPP. (G) Visual screen of effects of various concentrations of ATP and TPP on lysozyme phase behavior. All samples, except those for ζ-potential measurements, were prepared with 10 mg/mL lysozyme in 10 mM Tris buffer, pH 7.0. Samples for ζ-potential measurements contained 1 mg/mL lysozyme in 10 mM Tris buffer, pH 7.0.

For each polyphosphate, we prepared 28 lysozyme solutions with a range of polyphosphate concentrations for analysis. After 24 h or 60 days of incubation at room temperature, an aliquot of each solution was extracted, centrifuged to remove any solid precipitate, and the protein concentration in the supernatant was measured (Figures 1A,B and S1). After 24 h, protein concentration measurements show that the precipitation windows are similar for ATP and for TPP (∼1–25 mM). The drop in supernatant concentrations indicates a liquid–solid phase transition, resulting in a sufficiently large precipitate to obscure light. Visual inspection of samples indicates particles precipitated by TPP versus ATP are much larger (Figure 1G). After 60 days, we observed the formation of large protein crystals (Figure S2) in equilibrium with supernatants at a much lower protein concentration than that observed after 24 h (Figure 1a) for samples with ATP concentrations above 7.5 mM. In contrast, for TPP, a different crystal morphology was only observed in a single condition (40 mM) occurring along the resolubilization boundary (Figure 1B). The observed lower solubility of the protein crystal phase is consistent with phase diagrams of protein solutions, where gel phases or amorphous precipitates are metastable to the crystal.^37−40^

To probe the relationship between interactions and the phase diagram, the apparent mutual diffusion coefficient *D* in the supernatant was assessed by dynamic light scattering (DLS) (Figure 1C,D), and the net protein surface charge was assessed by the ζ-potential measurements (Figure 1E,F). The isoelectric point of proteins is defined as the pH at which the net charge of the protein is zero. At the experimental conditions used here (pH 7.0), binding of ATP/TPP to lysozyme reduces the net positive charge fixed on the protein surface and causes charge inversion at higher concentrations. With an initial increase of polyphosphate concentration, apparent values of *R*_h_ increase (or values of *D* decrease) due to screening repulsive electrostatics since *R*_h_ is smaller than the monomer value (1.9 nm). The increase of *R*_h_ above the monomer values indicates the formation of soluble protein clusters before precipitation occurs. To understand the protein–protein interactions underlying this behavior, the diffusion interaction parameter (*k*_D_) was estimated using eq 1, with this revealing that there are only minor differences in how ATP and TPP influence the overall nature of protein–protein interactions. In both cases, *k*_D_ values rapidly decrease to values near 0 mL/g in the salt concentration range of 0.3 to 1 mM which also corresponds to conditions where lysozyme charge is nearly neutral (see Figure 1E,F) indicating repulsive electrostatic interactions have been neutralized through polyphosphate binding. *k*_D_ values close to zero indicate the presence of weakly attractive interactions, which balance positive contributions from excluded volume forces and any residual electrostatic repulsion. The attractive forces are strong enough to induce precipitation, as reflected by the slight decrease in protein concentration of the supernatant above 0.3 mM salt concentration. The greatest amount of precipitation occurs over the salt concentration range of 1 to 10 mM, which coincides to a minimum in the *k*_D_ values, indicating solution-phase protein–protein attractions are the strongest. The attractions are weakened with a further increase in polyphosphate concentration above 10 mM, leading to protein resolubilization. This pattern of phase behavior and protein–protein interactions is referred to as re-entrant condensation, which has been studied in detail for solutions of acidic proteins in the presence of multivalent cations.^41−43^ For these systems, crystallization studies have shown multivalent ions cross-link acidic protein groups together causing the precipitation.^44−46^ In our previous study on lysozyme with TPP, we indirectly inferred an ion-bridging attraction because other multivalent anions such as citrate neutralized protein charge but did not induce strong enough attractions for precipitation to occur.^34^ The resolubilization region occurs when proteins become overcharged which is also evident here from considering the ζ-potential measurements shown in Figure 1E,F.^47^ While the initial precipitation behavior and solution-phase interactions are remarkably similar for ATP and TPP, there also exist significant differences. The precipitated phase in ATP has a different morphology and smaller particle size than for TPP, while crystallization occurs readily over time in ATP but not in TPP.

To investigate the protein-polyphosphate interactions which likely cause such notably different precipitation behavior, we examined these interactions further by X-ray crystallography and NMR spectroscopy.

### High-Resolution Structure of the Lysozyme–ATP Complex Reveals Three ATP Binding Sites on the Lysozyme Surface

To assess the binding sites of ATP and TPP to lysozyme and evaluate their binding mode, we attempted to co-crystalize lysozyme with various concentrations of ATP and TPP in a 10 mM Tris buffer, pH 7.0. Crystals of the lysozyme–TPP complex were obtained in a single condition, containing 40 mM TPP and diffracted X-rays at resolution above 4 Å in various space groups (*P*4_1_2_1_2, *P*422, and *P*12_1_2), with the data being severely anisotropic; therefore, further structure determination was not attempted.

Lysozyme–ATP crystals formed at various conditions (see Figure 1A). We acquired data on crystals formed at 10, 30, 50, and 80 mM ATP. They all diffracted X-rays at a similar resolution, with ATP molecules bound in the same binding sites, but the occupancy of the binding sites increased with increasing ATP concentration. We, therefore, used the data obtained on a crystal formed in condition with 80 mM ATP (protein/ATP = 1:53) for structure determination, which was solved to 1.27 Å resolution. Data collection and refinement statistics are shown in Table 1. The structure coordinates were submitted to the Protein Data Bank under accession ID 8AAZ. The lysozyme–ATP complex crystallized in the *P*4_3_2_1_2 space group, which represents ∼70% of the high-resolution crystal structures of hen egg white lysozyme reported in the PDB. ATP is more effective at producing highly ordered crystals, while TPP induces more anisotropic interactions, leading to various crystal forms.

**Table 1:** Crystallography Data Collection and Refinement Statistics for the HEWL–ATP Complex Structure

	HEWL–ATP complex^a^
Data Collection	
wavelength (Å)	0.98
space group	*P*4_3_2_1_2
Unit Cell Dimensions	
*a*, *b*, *c* (Å)	78.3893,78.3893, 38.114
α, β, γ (deg)	90, 90, 90
resolution range	35.06–1.27(1.315–1.27)
total reflections	1,644,783 (159,049)
unique reflections	31,905 (3125)
multiplicity	51.6 (50.9)
completeness (%)	99.99 (100.00)
*I*/σ*I*	50.22 (10.30)
Wilson *B*-factor (Ang.^2^)	12.22
*R*_merge_	0.0516 (0.2322)
*R*_meas_	0.05213 (0.2346)
*R*_pim_	0.007296 (0.03279)
CC1/2	1 (0.993)
CC*	1 (0.998)
Refinement	
reflections used in refinement	31,904 (3126)
reflections used for *R*-free	1998 (195)
*R*_work_	0.1395 (0.1243)
*R*_free_	0.1712 (0.1557)
*CC*_work_	0.965 (0.965)
*CC*_free_	0.955 (0.956)
number of non-hydrogen atoms	1298
macromolecules	1050
ligands	127
solvent	155
protein residues	129
R.M.S Deviations	
bond lengths (Å)	0.007
bond angles (deg)	1.09
Ramachandran	
favored (%)	99.21
allowed (%)	0.79
outliers (%)	0.00
rotamer outliers (%)	0.00
clashscore	3.16
B-Factors (Å^2^)	
average	20.96
macromolecules	15.69
ligands	57.55
solvent	34.72

a Statistics for the highest resolution shell are shown in parentheses.

**Figure 2:**
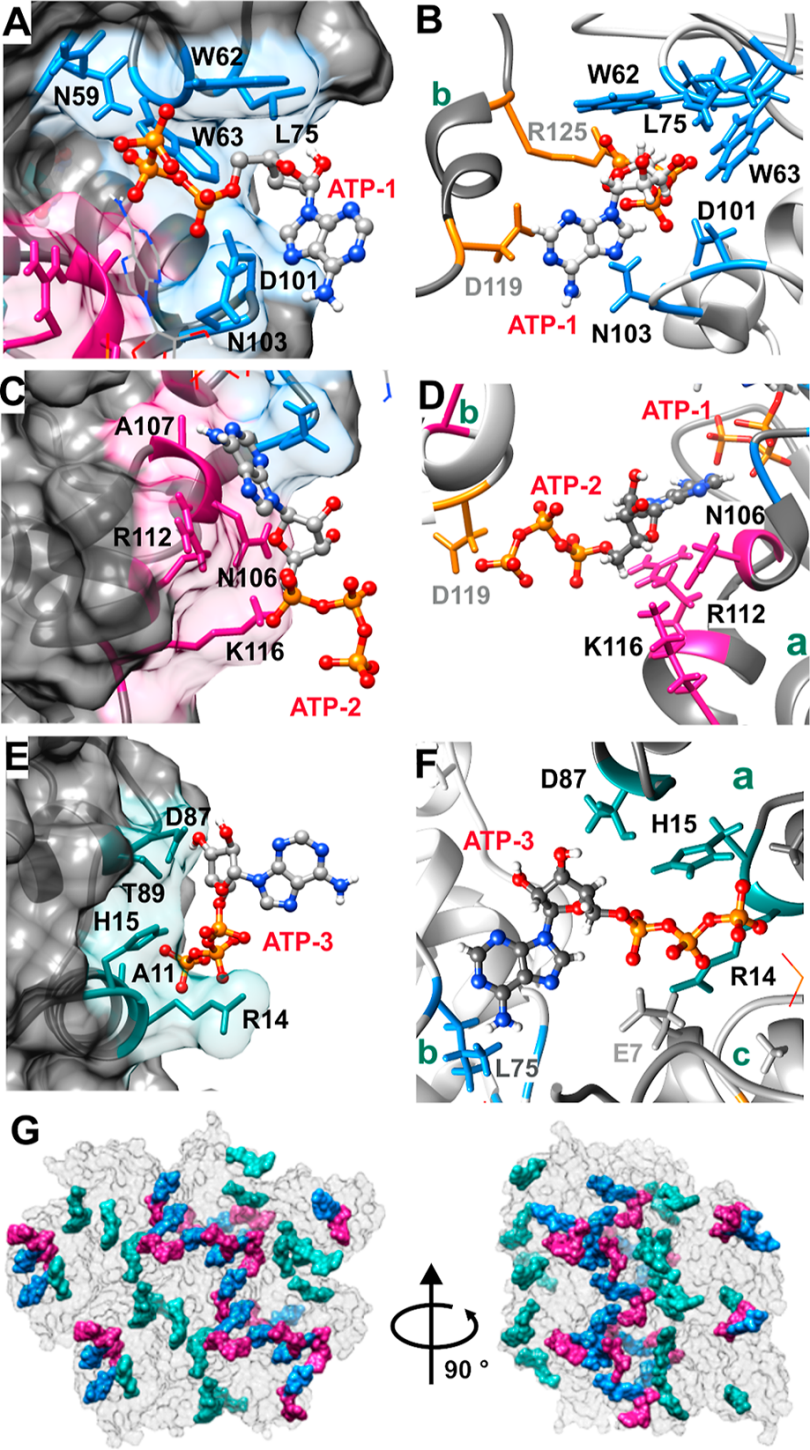
Structural characterization of the lysozyme and ATP interaction. (A) Binding site I (blue) with residues involved in ATP-1 binding depicted. (B) Interface between two proteins [dark gray (b) and light gray (a)] in crystal lattice where ATP-1 in site I interacts with D119 and R125 on the neighboring protein in the crystal lattice. (C) Binding site II (pink) with residues crucial for interaction with labeled ATP-2. (D) Interface between two proteins [dark gray (b) and light gray (a)] in the crystal lattice where ATP-2 bound to site II interacts with D119 on the neighboring protein b in the crystal lattice and with ATP-1 bound to the same protein molecule. (E) Binding site III (cyan) with residues crucial for interaction with labeled ATP-3. (F) Interface between three proteins (a–c) in the crystal lattice where ATP-3 in binding site III interacts with Leu75 on the first neighboring protein b and with E7 on the second neighboring protein c. (G) ATP crosslinking in the crystal lattice. A section of the crystal lattice is shown. Proteins are depicted in the surface representation (gray). Each lysozyme molecule has three ATP molecules bound to it: ATPs in binding sites I, II, and III are shown in blue, pink, and cyan, respectively. ATPs bound in sites I and II crosslink with each other, forming an ATP network that spans throughout the crystal lattice. The lysozyme–ATP crystals were obtained in a condition containing 10 mM Tris–HCl pH 7.0, 80 mM ATP-Na_2_ and 20 mg/mL protein at 294 K. Crystal structure of the lysozyme–ATP complex was solved at 1.27 Å resolution with 0.87, 0.98, and 0.79 occupancy of the ATP binding sites I, II, and III, respectively. The structure was deposited to PDB (ID 8AAZ). Residues involved in the ATP binding sites are shown in a stick representation and labeled accordingly.

The X-ray structure of the lysozyme–ATP complex revealed that there are three binding sites for ATP on the lysozyme surface. The first binding site (site I) coincides with the top of the lysozyme’s active cleft where Asn59, Trp62, and Trp63 residues interact with the tripolyphosphate moiety while Leu 75, Asp101, and Asn103 form additional contacts with the adenosine part of the ATP (Figure 2A). The second binding site (site II) consists of Asn106 and positively charged Arg112 and Lys116 that all interact with the tripolyphosphate moiety. It is important to note that the tripolyphosphate moiety of ATP bound in site I (ATP-1) interacts with the adenosine moiety of ATP bound in site II (ATP-2) via non-covalent interactions which further stabilize its binding (Figures 2B and S3). The third ATP binding site (site III) is formed of residues Arg14, His15, Asp87, and Thr89 that all interact with the tripolyphosphate moiety, while the adenosine ring is not further stabilized by any additional contacts (Figure 2C).

We further examined the crystal packing of the lysozyme–ATP complex. All three ATP molecules are located between the two proteins in the crystal lattice with the binding being asymmetric. In all cases, the crosslinking interaction occurs through the γ-phosphate group of ATP. In the crystal lattice, ATP in binding site I (ATP-1) interacts with Asp119 and Arg125 (binding site IV as identified by NMR below) on the neighboring lysozyme (Figure 2D). ATP in binding site II (ATP-2) forms contacts with Asp119 and ATP-1 bound on the neighboring protein (Figure 2E). ATP in binding site III (ATP-3) interacts with Leu75 and Asn77 on the symmetry neighbor protein molecule (Figure 2F). This data implies, that ATP drives protein cluster formation by bridging across two protein molecules, which in the case of lysozyme leads to crystal formation. It should be noted that ATP–ATP interactions between site I and site II lead to the formation of an ATP network that spans throughout the crystal lattice (Figure 2G). Dimers formed by ATP crosslinking interactions between two neighboring proteins are shown in Figure S4.

### ATP and TPP Bind to the Same Interaction Sites on the Protein Surface

While the crystal structure provided essential insights into ATP binding to lysozyme, the questions remain whether the observed binding occurs in solution as well, what are the binding constants for individual binding sites and whether TPP binds to lysozyme similarly to ATP. In order to explore these questions, we used NMR spectroscopy, which allows for the identification of interaction sites of small molecules on proteins at residue-level resolution and for the estimation of binding constants.

To identify binding sites for the polyphosphates on lysozyme, we acquired natural abundance ^13^C HSQC spectra for unlabeled lysozyme with increasing concentrations of ATP and TPP. To assess whether the binding occurs on the fast, intermediate, or slow exchange regime, we analyzed the halfwidth of lysozyme signals (Figure S5) during the course of the titration. We observed that the halfwidth of signals increases with increased ATP, which is consistent with the formation of small lysozyme–ATP clusters in solution, but no evidence of intermediate or slow exchange was identified. We, therefore, assumed that all the binding occured in the fast exchange regime.

**Figure 3:**
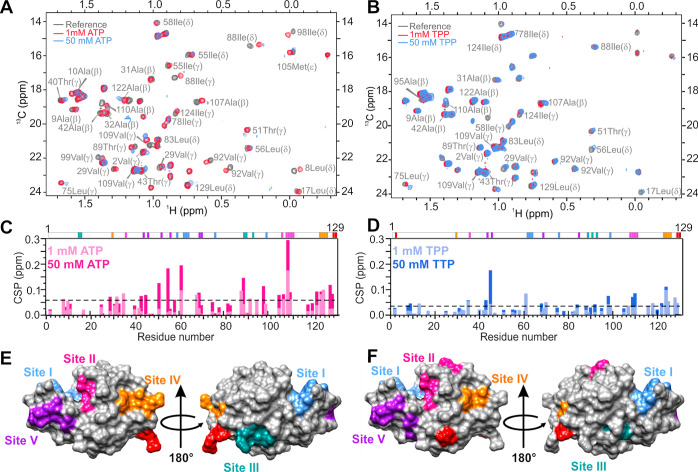
NMR evaluation of ATP and TPP interaction with lysozyme. (A) Overlay of the methyl region of ^13^C HSQC spectra of the lysozyme (gray) in the presence of 1 mM ATP (pink) and 50 mM ATP (blue). (B) Overlay of the methyl region of the ^13^C HSQC spectra of lysozyme (gray) in the presence of 1 mM TPP (pink) and 50 mM TPP (blue). ^13^C HSQC spectral assignments are based on BMRB ID 4562. (C) Maximum CSP in the methyl ^13^C HSQC spectra of lysozyme in the presence of 1 and 50 mM ATP. The threshold of significant CSP is 0.04 and is depicted with a black dashed line. Above the CSP plot, significantly perturbed residues are colored by the binding site. (D) Maximum CSP in the methyl ^13^C HSQC spectra of lysozyme in the presence of 1 and 50 mM TPP. The threshold of significant CSP is 0.04 and is depicted with a black dashed line. Above the CSP plot, significantly perturbed residues are colored by the binding site. (E) Residues with CSP above 0.04 at 50 mM ATP were mapped onto the lysozyme structure and colored by individual binding sites. (F) Residues with CSP above 0.04 at 50 mM TPP were mapped onto the lysozyme structure and colored by individual binding sites.

To determine the ATP interaction sites on the lysozyme surface, we calculated the CSP of individual residues with signals in the methyl region of the ^13^C HSQC spectra in the presence of 1 and 50 mM ATP (Figure 3A,C). These two concentrations were chosen as they lie on the precipitation (1 mM) and resolubilization (50 mM) boundaries, while the lysozyme concentration in the soluble fraction is still sufficient to acquire good-quality NMR spectra in a timely fashion. In the case of ATP, we acquired additional spectra of good quality for solutions containing up to 3 mM ATP and calculated the concentration-dependent chemical shift perturbations (CSP) (Figure S6). At ATP concentrations higher than 3 mM, the spectral quality deteriorated due to the precipitation of the protein and only improved upon protein resolubilization at high ATP concentrations.

The threshold value of significant CSPs was calculated using the Schumann method^48^ and was 0.04 for ATP. For TPP, the Schumann method did not produce reliable results due to a small CSP. Therefore, the same value as for ATP was used as a threshold in TPP studies. The residues were designated as interacting with ATP if the corresponding CSP at 50 mM was above the threshold value either in the ^13^C HSQC or ^1^H spectra (Figure 3C). Using this method, we determined six binding sites for ATP on the lysozyme surface (Figure 3E and Table S1). Binding site I (blue) consists of Arg61, Trp62, and Trp63; binding site II (pink) includes Ala107, Trp108, Val109, and Ala110; while Ile88, Thr89, and Val92 make up binding site III (dark cyan). These three binding sites are the same as the binding sites identified in the crystal structure of the ATP–lysozyme complex. Three additional binding sites were identified by NMR: binding site IV (orange) includes Trp123, Ile124, and Arg125; binding site V (purple) Arg68 and Thr69; and binding site VI (red) Arg128 and Leu129. Binding site IV is also present in the crystal lattice where ATP-1 is located between two protein molecules packed together (see Figure 2B). Residues in binding sites V and VI are involved in protein–protein contacts crucial for crystal packing and are thus not available for ATP binding when lysozyme is crystallized.

In the case of TPP, protein precipitation prevented the acquisition of good-quality spectra above 1 mM of TPP until sufficient resolubilization was achieved at 50 mM. Therefore, the TPP concentration dependence of CSPs could not be determined. However, we could estimate the TPP binding sites on the lysozyme surface by comparing the CSP data in the presence of 1 mM and 50 mM TPP to that obtained by ATP. Overlay of the methyl region of the ^13^C HSQC spectra revealed that, similarly to ATP, TPP binds to the protein surface in a site-specific manner that is also concentration-dependent (Figure 3B,D). In general, ATP induces a larger CSP than TPP, likely due to the interactions between the adenosine part of ATP and the side chains of amino acids on the lysozyme surface. It should be, however, noted that binding of TPP, even if it occurs at the same binding affinity as ATP, results in a smaller CSP due to the absence of ring current shifts in TPP. The threshold value of significant CSPs was calculated as described above. Plotting of the CSP against the protein sequence shows that both ATP and TPP bind to the same binding sites, that include both charged, polar and nonpolar amino acids (Figure 3C−F).

**Figure 4:**
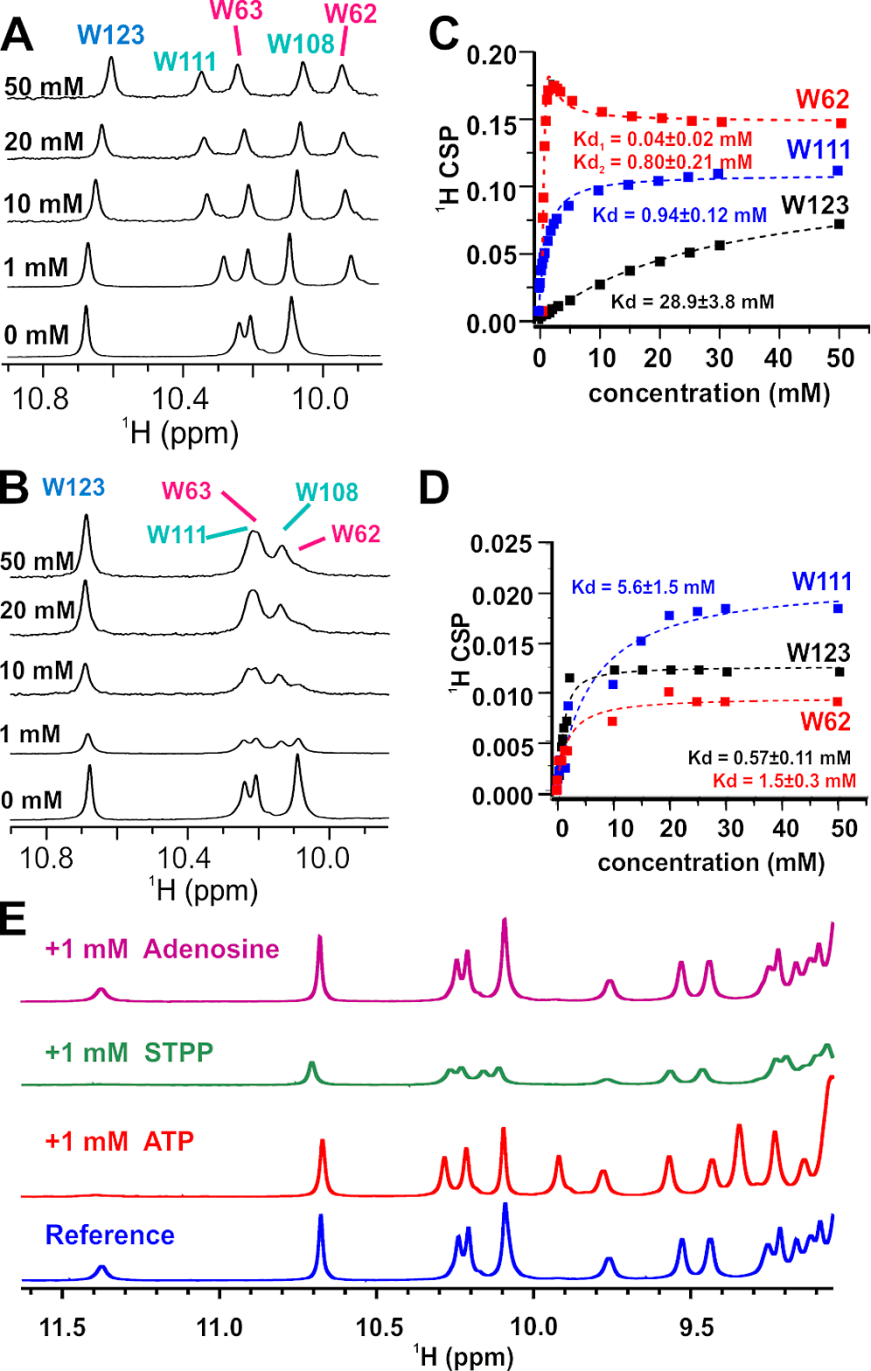
^1^H NMR evaluation of ATP/TPP–lysozyme interaction. (A) Stack of imino regions of ^1^H spectra of lysozyme in the presence of various concentrations of ATP showing CSP of imino protons. Annotation of residues is shown above signals. (B) Stack of imino regions of ^1^H spectra of lysozyme in the presence of various concentration of TPP showing CSP of the imino protons. Annotation of residues is shown above signals. (C) Apparent binding affinities for ATP–lysozyme interaction were estimated from the proton CSP of the tryptophan signals located within individual binding sites. W62 signal was affected by two binding events and was fitted to a 2:1 binding model. (D) Apparent binding affinities for the TPP–lysozyme interaction were estimated from the proton CSP of the tryptophan signals located within individual binding sites. (E) Comparison of the imino and aromatic regions of the reference (blue) lysozyme ^1^H spectra and lysozyme spectra in the presence of 1 mM ATP (red), 1 mM TPP (green), and 1 mM adenosine (purple).

While concentration-dependent CSPs and estimation of ATP binding constants were possible for some of the identified binding sites, such an approach could not be utilized for TPP, where protein precipitation and the subsequent decrease in the natural abundance of ^13^C HSQC spectra quality prevented the determination of binding constants. However, the identification of binding sites revealed that there are tryptophan residues either within or in the vicinity of the polyphosphate binding sites for three of the six individual sites, which enabled us to estimate apparent *K*_d_ values for these sites. W62 corresponds to the binding site I, W111 to the binding site II, and W123 to the binding site IV. The ^1^H CSPs data of the W111 and W123 imino protons (Figure 4) was fitted to a one-binding site model, while the data for W62 was fitted to a two-binding site model to account for the non-monotonic behavior. For TPP, the binding site model provided a good fit for all three of the sites (I, II, and IV). Table S1 also contains estimated *K*_d_ values for ATP interacting with sites III, V, and VI from fitting to the ^13^C HSQC data

Our data show that ATP and TPP interact with the same sites located on lysozyme (Table S1). All binding sites on lysozyme exhibit similar binding affinities for TPP (∼1 mM). While ATP also binds with millimolar affinity to sites II, III, and V, remarkably different binding is observed for sites I and IV, with affinities being sub-millimolar for site I and greater than 10 mM for site IV. To gain further insight into the binding mode of ATP and TPP to lysozyme and to determine whether the adenosine ring or the tripolyphosphate determines the interaction with the protein surface, we compared the ^1^H NMR spectra of lysozyme in the presence of ATP, TPP, and adenosine (Figure 4E). While ATP and TPP clearly interact with lysozyme, no CSP was observed when adenosine was added to the protein, indicating that adenosine does not interact with the protein on its own. This further implies that it is the tripolyphosphate chain of the ATP that is crucial for the interaction with the protein, while the adenosine ring likely stabilizes the interactions through the π–cation interactions and thus contributes to the binding affinity of ATP, as demonstrated in the case of binding to site I.

### ATP and TPP Preferentially Interact with Arginine Residues

**Figure 5:**
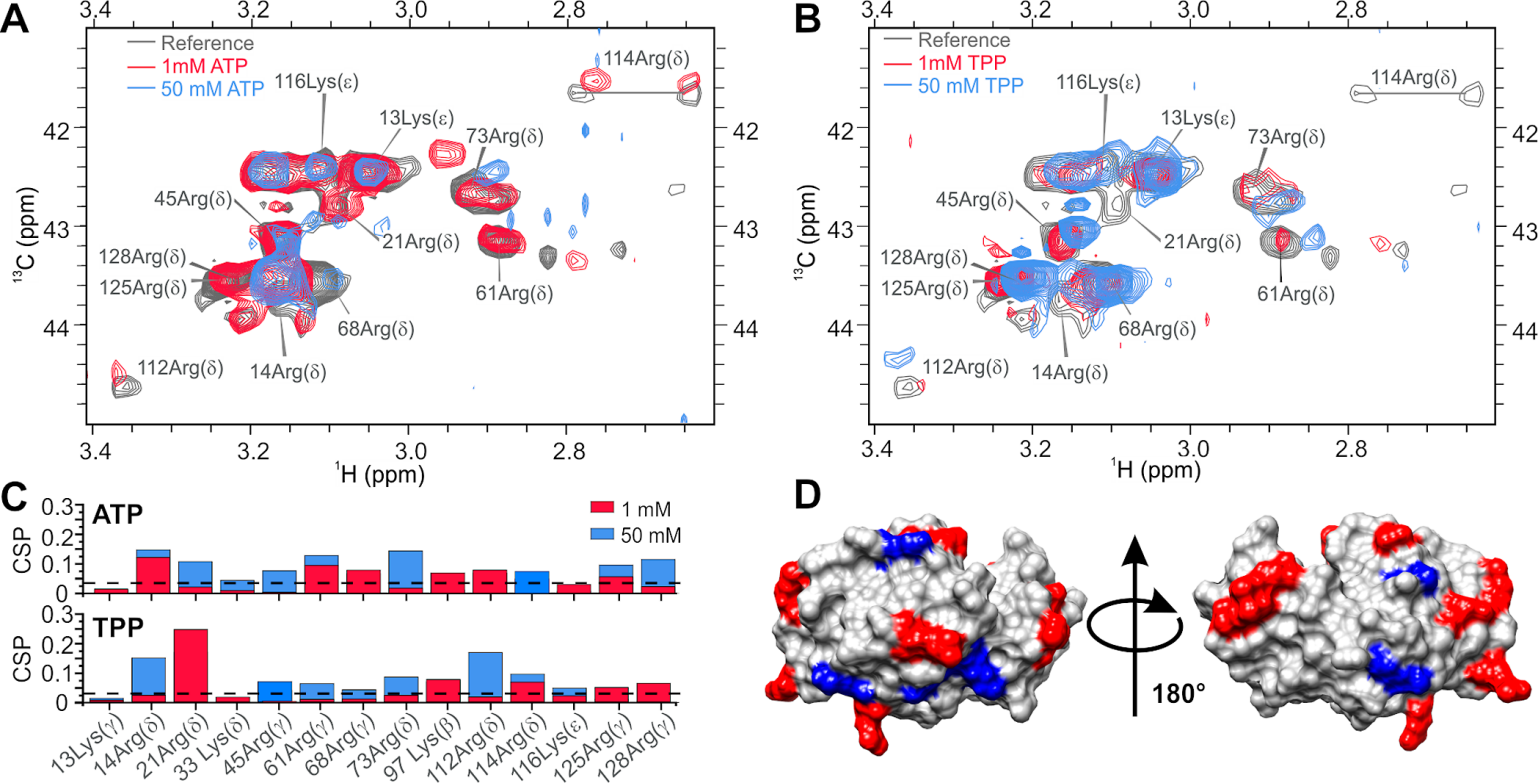
NMR evaluation of ATP and TPP interaction with arginine and lysine residues (A) Overlay of the ^13^C HSQC spectra of lysozyme (gray) in the presence of 1 mM ATP (pink) and 50 mM ATP (blue). (B) Overlay of the ^13^C HSQC spectra of lysozyme (gray) in the presence of 1 mM TPP (pink) and 50 mM TPP (blue) ^13^C HSQC spectral assignments are based on BMRB ID 4562. (C) CSP in the ^13^C HSQC spectra of lysozyme of Arg and Lys residues in the presence of 1 and 50 mM ATP (top) and TPP (bottom). The threshold of significant CSP is 0.04 and is depicted with a black dashed line. (D) Distribution of lysine (blue) and arginine (red) residues on the lysozyme surface.

At pH 7.0, which was used in this study, five amino acids contribute to the overall charge of the proteins: Asp and Glu are negatively charged, Arg and Lys are positively charged, while his positive charge depends on the local environment. In the experimental conditions used here, lysozyme has a positive charge that is largely dominated by arginine (11) and lysine (6) residues that are evenly distributed around its surface (Figure 5D). To gain insight into whether the polyphosphates used in this study show any preferential binding to Lys or Arg residues, we analyzed the natural abundance ^13^C HSQC spectra containing side chain C–H correlations of Arg and Lys signals (Figure 5A,B). For each residue, we chose a single peak that showed no or minimal signal overlap for further analysis. Ten out of twelve arginine residues and four out of six lysine residues were unambiguously assigned. The CSP analysis shows that the majority of CSP are concentration-dependent, except where the binding sites are already saturated at 1 mM of the polyphosphates (Figure 5C).

Arginine and lysine residues are mostly located in the vicinity of each other and are evenly distributed on the lysozyme surface (Figure 5D). Using crystallography and NMR, we established that Arg residues are involved in all six identified binding sites, whereas only site I contains a Lys residue (Table S1). A closer inspection of ATP/TPP interactions with Arg and Lys residues (Figure 5C) revealed that all Arg but only two Lys residues interact with ATP and TPP at high concentrations. Additionally, for solvent-exposed arginine and lysine pairs located proximal to each other (Figure S7): Lys13-Arg14, Lys33-Arg114, and Lys13-Arg125-Arg128 we observe that polyphosphates interact with arginine but not with lysine residue. This data clearly shows that both ATP and TPP preferentially bind to arginine over lysine residues. This is in line with recent reports that ATP preferentially interacts with arginine residues on the protein surfaces, and Arg to Lys mutations impede these interactions.^25,29,49^

### ATP Binding to Individual Binding Sites Is Not Affected by the Presence of Mg^2+^ Ions

Using ^31^P NMR, we established that a fully saturated ATP–Mg complex forms at equimolar concentrations of ATP and MgCl_2_ (Figure S8), with Mg^2+^ predominantly complexed between β and γ phosphate groups of ATP, which is in line with previous reports.^50^ Hence, an equimolar mixture of ATP and MgCl_2_ was used to assess the effects of ATP complexation with Mg^2+^ on binding to lysozyme. TPP, on the other hand, forms an insoluble complex with Mg^2+^ even at low concentrations; therefore, further experiments using TPP–Mg were not performed.

**Figure 6:**
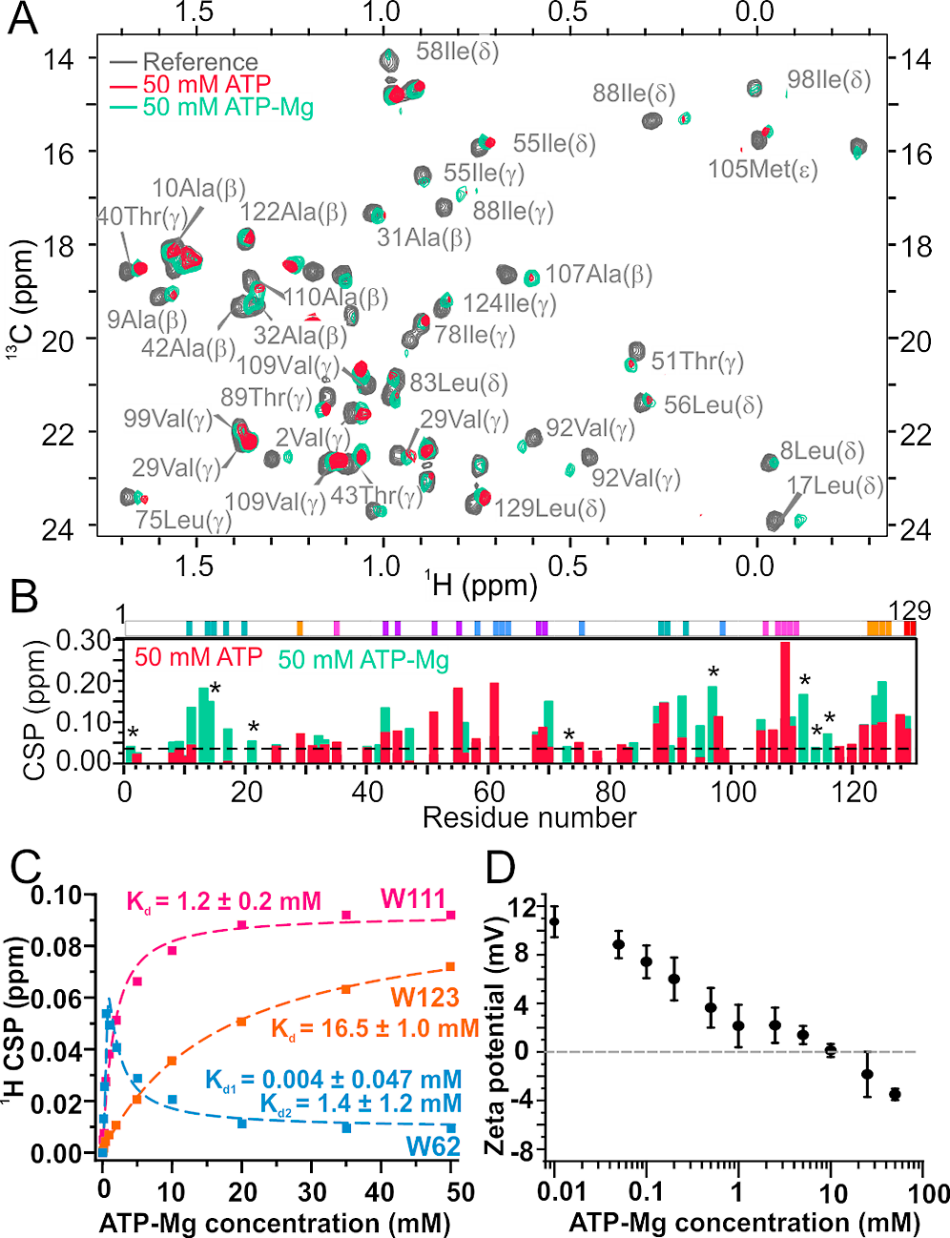
NMR evaluation of ATP–Mg interaction with lysozyme. (A) Overlay of the methyl region of the ^13^C HSQC spectra of lysozyme (gray) in the presence of 50 mM ATP (pink) and 50 mM ATP–Mg (green). ^13^C HSQC spectral assignments are based on BMRB ID 4562. (B) Maximum CSP in the methyl ^13^C HSQC spectra of lysozyme in the presence of 50 mM ATP and 50 mM ATP–Mg. The threshold of significant CSP is 0.04 and is depicted with a black dashed line. Signals that were not observed in the 50 mM ATP spectra due to protein precipitation but are visible in the 50 mM ATP–Mg are denoted with an asterisk (*). Above the CSP plot, significantly perturbed residues are colored by the binding site. (C) Apparent binding affinities for ATP–Mg and lysozyme interactions were estimated from the proton CSP of the tryptophan signals located within individual binding sites. W62 signal was affected by two binding events and was fitted to a 2:1 binding model. (D) ζ-Potentials for lysozyme at various concentrations of ATP–Mg. All samples were prepared with 1 mg/mL lysozyme in 10 mM Tris buffer, pH 7.0.

To identify binding sites of the ATP–Mg on lysozyme, we acquired natural abundance ^13^C HSQC spectra for unlabeled lysozyme with increasing concentrations of ATP–Mg. In contrast to ATP alone, ATP–Mg did not induce precipitation of lysozyme, so that good quality spectra could be obtained up to 50 mM ATP–Mg, while at higher concentrations, the signal intensity decreased too much to obtain reliable data due to the effect of high ionic strength on NMR probe sensitivity.^51^ Comparison of the concentration-dependent CSP data of lysozyme in the presence of an increasing concentration of ATP–Mg to that obtained for ATP (Figure 6A,B) revealed that ATP–Mg binds to the same six binding sites as ATP (Figures 6C and S10 and Table S1). Furthermore, the affinities of ATP and ATP–Mg for each of the sites are of similar magnitude, which is not surprising since the amino acid compositions in each of the binding sites are identical to each other. The only exceptions are an additional residue involved in sites II, III, and VI, that was not observed in the presence of ATP alone due to protein precipitation (Figure 6B and Table S1). Additionally, we also show that the binding of ATP is not affected by the addition of up to 200 mM NaCl (Figure S11), which shows that ATP can interact non-specifically with proteins in the cell environment.

ζ-potential measurements revealed that ATP–Mg still overcharges lysozyme, but at much higher concentrations than ATP on its own (∼10 mM ATP–Mg to reach net charge 0 mV compared to ∼1 mM ATP, Figure 6D). This confirms that Mg^2+^ ions are not released from the ATP–Mg complex prior to ATP interacting with lysozyme. However, the inability to precipitate lysozyme indicates the complexing of ATP with Mg^2+^, which reduces its ability to form ion bridging attractions through the polyphosphate moiety.

## Discussion

Recently, it has been established that both ATP and TPP can specifically or non-specifically interact with intrinsically disordered or globular proteins and that they can influence their aggregation, fibrillation, or phase separation behavior. However, only limited data is available on the nature of non-specific ATP interaction sites on protein surfaces, and the mechanism by which ATP influences protein self-assembly is largely unknown. In this study, we evaluated in detail the ATP interactions with a model protein, hen egg-white lysozyme, using X-ray crystallography and NMR and studied their effect on lysozyme protein–protein interactions and phase behavior by DLS. We also compared the interactions of ATP and TPP with the lysozyme surface and evaluated how they influence the crystallization of lysozyme.

### Ambiguous Nature of Polyphosphate Interaction Sites

Lysozyme has been extensively studied as a model protein for studying the effects of anion binding on protein crystallization.^52−57^ Multiple binding sites for anions have been identified on its surface; some of these sites are lysozyme-specific, while others are space-group and anion specific.^56^ Lysozyme is also known to bind DNA and RNA both specifically and non-specifically,^58,59^ but has no known specific binding sites for ATP. Here, we identified three ATP molecules bound to lysozyme in an asymmetric unit of the crystal structure. Binding site I is unique for ATP and has not previously been identified as an anion binding site in other published structures but is a suspected DNA binding site.^58,59^ Site II and site III have been observed previously for other mono- and multivalent anions; site II for NO_3_^–^ and I^–^,^56^ while site III is a known PO_4_^3–^ and SO_4_^2–^ binding site.^53^

The same three binding sites were also observed using NMR, which identified three additional non-specific ATP binding sites on the lysozyme surface. A recent computational study of ATP clustering around the lysozyme surface reported predictions of the ATP-susceptible regions in the lysozyme sequence^33^ that are in good agreement with our experimental results with one important exception (Figure S12). In this particular study, ATP clustering around lysozyme identified a relatively continuous and flexible ATP-binding region between Lys33 and Thr69. While we observed strong binding in the Arg61-Arg68 region (site I), we did not experimentally detect any binding in the Lys33-Ser60 region (except for non-specific interaction with Arg45 at high ATP concentrations). This further implies that flexibility is not as significant for non-specific ATP binding as proposed by the molecular simulations and suggests that experimentally generated data should be used as a starting point for developing better models for capturing non-specific interactions of ATP with proteins.

ATP and TPP interact with the same sites on the protein surface, but binding affinities for individual sites may vary between the two. Identified binding sites for ATP can be divided into two categories according to the strength of the affinity: there is one strong binding site (site I) with sub 50 μM affinity, while all other binding interactions have affinities of millimolar range or greater. In site I, the triphosphate moiety forms a non-covalent interaction with aromatic residues and is the only site where an adenosine group interacts with the protein surface. This is the reason why the *K*_d_ value is much smaller than the corresponding value for TPP, which is around 1 mM. According to the crystal structure, the only other interaction involving adenosine is between ATP-2 and the triphosphate of ATP-1. Indeed, the binding affinity of ATP is also slightly stronger than that of TPP in site II.

TPP measurements for sites I, II, and IV (see Figure 4D) indicate millimolar binding affinities can be achieved through interactions of the triphosphate moiety with protein surface groups without any further stabilization of the adenosine group. Indeed, for site III, no adenosine interactions with the protein surface are observed in the ATP–protein crystal, while the *K*_d_ for ATP binding is ∼1 mM. Interestingly, for site IV, the binding constant is weaker for ATP than for TPP. However, the HSQC data indicate all other protein groups involved in binding at site IV exhibit millimolar affinities, suggesting that there is something anomalous about the W123 and that the ATP affinity for site IV is more similar to the value observed for TPP around 1 mM. As such, while binding affinities for TPP in sites III, V, and VI could not be determined, we suspect similar strengths of interaction with the protein for TPP and ATP, where the predominant mode of interaction occurs through phosphate groups. This is also evident from overlaying the precipitation boundaries (Figure 1A,B), protein–protein interaction (Figure 1C,D), and ζ-potential profiles (Figure 1E,F) for ATP versus TPP and from the almost identical effectiveness of ATP and TPP at preventing the aggregation of globular proteins under thermal stress.^27,28^

Our results indicate the nature of ATP binding to folded protein surfaces is markedly different from binding to IDPs and IDRs; for folded protein surfaces, the TPP moiety determines the non-specific interaction, and only for the specific binding site on lysozyme (site I) is there a difference between ATP and TPP affinities. On the other hand, there are more significant differences between TPP and ATP binding to IDPs and IDRs with the adenosine ring increasing the specificity and affinity of these interactions.^22^ Additionally, our results show that arginines could be more important for interactions on folded surfaces than with IDRs that are richer in lysine and aromatic residues.

It is tempting to rationalize the biphasic binding behavior observed for W62 from knowledge of the proximal locations of bound ATP molecules in the protein crystal. ATP in site I interacts with the ATP in site II, while the binding constant for site II is within the range of uncertainty equal to the weak binding constant estimated for site I (*K*_d,2_ in Figure 4C). Taken together, this suggests the biphasic behavior occurs due to site I binding an ATP molecule, where the configuration of the bound ATP changes when another ATP molecule is bound at site II. Consistent with this hypothesis, the biphasic behavior with TPP does not occur because there is no possible interaction between a pair of TPP molecules bound in sites I and II.

### ATP but Not TPP Is an Effective Crystallization Agent

Here we report a high-resolution crystal structure of the lysozyme–ATP complex, where the crystals were obtained by co-crystallization of both molecules. The space group observed in all tested lysozyme–ATP crystals was *P*4_3_2_1_2, which is the most common space group reported for high-resolution crystal structures of hen egg white lysozyme in the PDB and is the same space group reported for lysozyme crystallized in the presence of NaCl.^56^ This indicates that ATP does not cause crystallization by forming new crystal contacts, as has been observed with trivalent cations, but rather increases the crystal stability through cross-linking groups together that are already proximally located in the crystal without disrupting other pre-existing crystal contacts. In contrast, the lysozyme–TPP complex crystalized in a number of space groups, including *P*4_1_2_1_2, *P*422, and P12_1_2, which are more rarely observed space groups of lysozyme structures. Such behavior was previously reported for monovalent anions, such as Br^–^, I^–^ NO_3_^–^, where anion binding induces the formation of new crystal contacts causing a change to the crystal packing space group from tetragonal to orthorhombic *P*2_1_2_1_2_1_, monoclinic *P*2_1_, or triclinic *P*1,^56^ while multivalent ions like SO_4_^2–^ and PO_4_^3–^ were shown to co-crystalize with lysozyme in both tetragonal *P*4_3_22, and orthorhombic *P*2_1_2_1_2_1_ space groups, although only the orthorhombic lysozyme structures were deposited to PDB.^53^ This suggests that TPP binding is required for stabilizing crystal contacts through the formation of cross-linking interactions, which would otherwise not form in the absence of TPP. Interestingly, none of these space groups correspond to that of the ATP co-crystal (*P*4_3_2_1_2), even though the majority of the ATP-mediated cross-linking interactions occur through hydrogen bonds involving phosphate groups. These interactions can also be formed by TPP, which is reflected by the high binding affinity of TPP to sites I, II, and IV.

The key question to address is why ATP but not TPP is an effective crystallization agent, as the phosphate groups appear to mediate most of the cross-bridging interactions. The measured solubilities of the ATP–lysozyme crystal are much lower than values reported in the literature for a range of salt precipitants,^53,54,60^ indicating that ATP induces energetically favorable contacts in the protein crystal, while the ease of crystallizing lysozyme in ATP suggests that the activation barrier has been sufficiently reduced relative to solutions with TPP. We only identified two significant crystal-phase interactions formed by the adenosine group in sites I and II. NMR data also indicate much stronger binding of ATP than TPP to site I and to a lesser extent to site II. This suggests the ATP molecule bridging together sites I and IV, which occurs near a crystal contact, provides the dominant energetic contribution to the increased crystal stability of the ATP–protein complex. Because binding of ATP at site IV is also detected by NMR, it is likely ATP causes lysozyme to self-associate through sites I and IV in solution, which could explain the enhanced crystallization kinetics. It is not physically unrealistic that the formation of a single protein–protein contact can have such dramatic consequences on protein crystallization behavior. Single point mutations to human γD-crystallin (HGD), which cause large changes to crystal solubility and nucleation kinetics^61^ and alter the temperature-dependent solubility behavior from normal to retrograde,^62^ have been attributed to introducing an energetically-favorable anisotropic interaction. Indeed, this principle is relied upon in strategies to improve protein crystallizability through introducing single-point mutations.^63−65^ Our data suggest ATP-induced lysozyme self-association in solution leads to the formation of transient clusters containing orientationally constrained lysozyme molecules in pre-crystalline configurations, which reduces the entropic barrier to crystallization, leading to the enhanced nucleation.

Another possible way ATP could increase crystallization rates is through altering the nucleation pathway to proceed through a two-step mechanism.^66−68^ In this case, the nucleation barrier is reduced through the formation of a so-called metastable intermediate phase (MIP), which corresponds to clusters or aggregates of proteins with liquid-like order. The second step in the nucleation pathway corresponds to rearrangements of proteins within the MIPs to give crystalline-like order. This type of mechanism has been used to explain enhanced nucleation rates of proteins in regions of the phase diagram which are proximal to the liquid–liquid critical point or the dilute branch of the gas–liquid binodal. For solutions with trivalent cations, the two-step mechanism has been directly observed using real-time SAXS studies to show the crystals evolve directly from the MIPs.^69^ However, in contrast to our phase behavior studies, the enhanced crystallization only occurs at the precipitation boundaries.^45,46,69^ At salt concentrations within the precipitated region, crystallization does not occur because protein–protein interactions are too attractive, causing protein molecules to be trapped in disordered precipitates where local rearrangements are not possible. This behavior reflects the crystallization window discovered by George and Wilson,^70^ which requires that protein–protein interactions are only moderately attractive in order for crystallization to occur. In contrast, for ATP-containing solutions, crystallization occurs readily throughout the precipitation region, even though the strengths of protein–protein attraction are expected to be as strong, if not stronger, than occurs in solutions of acidic proteins with multivalent cations.^34^ For TPP, we showed that the precipitated region corresponds to solution conditions where protein–protein interactions are more attractive than specified by the crystallization window. For ATP, the crystal solubility measurements of less than 0.02 g/L provide an indication that the corresponding region of the phase diagram is also outside the crystallization window. As such, determining how ATP crystallizes proteins has significant implications toward a more general understanding of factors controlling protein crystallization.

### Implications for Globular Protein Solubility

Previous work has suggested proteins are maintained in a dispersed state through ATP binding to flexible regions, which prevents their local unfolding to expose hot spots.^32^ Interestingly, we do not observe any strong binding of ATP to the flexible loop region of lysozyme, indicating that other routes for stabilization by ATP might exist. Indeed, if stabilization by ATP occurs by inhibition of local unfolding, aggregation propensity should correlate with melting temperature as ATP concentration is varied. In our previous work, examining the effect of ATP on aggregation of ovalbumin upon thermal stress is suppressed in the concentration range of 1 to 20 mM ATP,^28^ while the melting temperature changes between 0 and 1 mM and at concentrations above 50 mM ATP.^27,28^ Rather, protein–protein interaction and ζ-potential measurements provided evidence that aggregate growth was suppressed through supercharging proteins by binding ATP to increase their colloidal stability at concentrations as low as 1 mM. A similar pattern of protein aggregation with respect to ATP and TPP concentrations has been observed for bovine serum albumin (BSA).^27,28^ The behavior provides indirect evidence that ATP molecules bind to ovalbumin and BSA with millimolar affinity, which is a similar magnitude to the measured binding for ATP to lysozyme. This suggests that ATP binding to proteins is not sensitive to the overall net charge on the protein, as ovalbumin and BSA are acidic proteins while lysozyme is basic. Indeed, in the SI, we report ^1^H and ^13^C HSQC measurements to another acidic protein, human serum albumin (HSA) (Figure S13), which provides direct evidence that ATP is binding non-specifically at concentrations as low as 1 mM.

It is unlikely that overcharging effects are significant in physiological conditions since electrostatic interactions become less significant at ionic strengths greater than 100 mM. However, Wennerstrom et al.^70^ have argued that the electrostatic screening length is much greater than expected in cellular environments because most small anions are not free but rather bound in macromolecular complexes. On the other hand, in solutions with equimolar concentrations of Mg^2+^ and ATP, which resemble cellular environments, overcharging effects are suppressed due to the formation of ATP–Mg complexes (see Figure 6D).

An important result of our study is that ATP and TPP preferentially bind to arginine groups. This finding is supported by molecular simulations of lysozyme^49^ and closely follows the polyphosphate ion binding propensity for IDR regions.^17,25,26,29^ Given that ATP strongly interacts with arginine groups irrespective of protein disorder or flexibility, there may also be similarities in how ATP modifies solubility of IDPs compared to folded proteins. The increased insolubility and phase separation tendency of IDPs has been attributed in part due to the stickiness of arginine, which arises from the ability to form cation−π and π–π interactions most commonly with aromatic groups. This has been demonstrated by mutation studies in which the substitution of all arginines by lysines abolishes the ability of IDR-containing domains to form LLPS.^71−73^ Furthermore, a proteome-wide study found that arginine-to-lysine content correlated better with IDR insolubility than some commonly used hydrophobicity and aggregation/amyloidogenicity scales.^74^ It is likely that arginine stickiness is greatly reduced upon binding to ATP, as evidenced by studies showing that ATP solubilizes the N-terminal domain of FUS and the prion-like domain of TDP-43 through direct binding interactions with arginine-containing IDR regions.^22,25^ That the effects of ATP occur more broadly for IDRs is consistent with ATP’s ability to solubilize 25% of the human proteome, with most solubilized proteins being rich in basic versus acidic amino acids.^20^ Arginine-aromatic group interactions are also major factors contributing to the stability of folded proteins^75,76^ and protein–protein complexes,^77^ while arginine to lysine content correlates with the insolubility in the *E. coli* proteome.^78^ Given the ubiquitous nature of arginine-mediated interactions, we expect them to play a role in determining the aggregation propensity of many folded proteins. This was proven by a study of a series of single- and multi-point arginine–lysine swap mutants for a scFv domain protein. Exchanging lysine for arginine increased the tendency of partially folded and unfolded states to associate but did not alter the association of the native state.^79^ While the structural factors causing aggregation could not be isolated directly, the results provided indirect evidence that aggregation was caused by arginine interacting with aromatic groups exposed when the scFv partially unfolds. Taken together these findings do suggest protein aggregation propensity is enhanced by arginine-aromatic interactions, which could be targeted and prevented through binding ATP. Consistent with this hypothesis, ATP is effective at suppressing aggregation upon thermal stress for lysozyme, ovalbumin, BSA, and ribonuclease A, but not α-chymotrypinogen (α-Cgn), which is the only protein of the group with a low arginine content.^18,27,28^

## Conclusions

In summary, we performed a comprehensive structural study of ATP and TPP interaction with lysozyme combining crystallography, NMR, and light scattering. Here, we have identified many more non-specific binding sites of ATP with millimolar affinities than previously observed in NMR studies of non-specific interactions of ATP with model proteins^32^ and arginine-rich crystallins.^31^ While previous studies focused on detecting the interactions using protein backbone observed experiments, we employed side-chain cross-peak correlations to detect these interactions. We determined six well-defined binding sites for ATP and TPP on lysozyme and identified further non-specific interactions with other Arg residues on the lysozyme surface at high ATP and TPP concentrations. We show protein binding affinities for TPP and ATP are of similar strength (∼1 mM), with the exception of site I (*K*_d_ < 100 μM), which is partially stabilized by interactions involving the adenosine group. It is clear that the TPP moiety of ATP drives the interaction with the protein surface, and only a smaller subset of binding interactions involves the adenosine group. While the non-specific interactions involving phosphate groups determine the impact of ATP on solution-phase protein–protein interactions and lysozyme electrostatic properties, the highly directional cross-linking interaction of ATP between site I and site IV is crucial for lysozyme crystallization.

We expect such non-specific interactions of ATP to occur with both acidic and basic proteins and play a significant role in cellular environments where ATP concentrations are in the millimolar range (∼4 mM on average), which is much higher than the micromolar concentrations needed for cellular function.^80^ Indeed, we show that in a cellular-like environment, with magnesium and sodium ions at physiological concentrations, ATP non-specifically interacts with globular proteins. ATP is a highly charged molecule; therefore, its binding to proteins significantly alters their surface properties that can in turn impact their self-assembly and phase behavior. As such, this finding has further implications for understanding the role of ATP in maintaining the colloidal stability of folded proteins inside the cell, where an intricate interplay between attractive forces needed for protein recognition and normal cellular function and repulsive forces that keep the proteins dispersed is needed to maintain cellular homeostasis.^81^

## Experimental Procedures

### Sample Preparation

Chicken egg lysozyme with a purity of ≥98%, Tris base and ATP with a purity ≥99% were purchased from Sigma-Aldrich (Sigma-Aldrich, Gillingham, UK). Sodium tripolyphosphate (TPP) with a purity ≥99% was sourced from Fisher Scientific UK Ltd. (Fisher Scientific, Loughborough, UK).

All buffer solutions were prepared volumetrically and filtered with a 0.1 μm hydrophilic nylon membrane (Merck Millipore Ltd., Ireland) before use. Stock solution of lysozyme was prepared by dissolving the protein in 10 mM Tris buffer, pH 7.0 and then dialyzed against 600 mL of the buffer solution for 4 h twice and again overnight. After dialysis, the pH of the lysozyme stock solution was checked again and adjusted to pH 7.0 (±0.05) if needed, and the protein stock concentration was adjusted to 20 mg/mL. Finally, the lysozyme stock solution was filtered through a series of 0.22, 0.1, and 0.02 μm hydrophilic nylon membranes (Merck Millipore Ltd., Ireland). Protein concentrations were determined by measuring UV absorption at 280 nm using NanoDrop 2000 (Thermo Fisher Scientific). 0.4 M ATP and 0.4 M TPP stock solutions were prepared in 10 mM Tris buffer pH 7.0, pH adjusted if needed, and filtered through a 0.1 μm hydrophilic nylon membrane (Merck Millipore Ltd., Ireland) before use.

### Protein Precipitation Measurements

For protein precipitation studies, a series of 10 mg/mL lysozyme samples and ATP or TPP concentrations ranging from 0 to 100 mM in 10 mM Tris pH 7.0 were prepared. After mixing, the samples were left to equilibrate at room temperature for 60 days. After 2 h of incubation, 50 μL of supernatant was collected, centrifuged at 10,000 rpm for 10 min using a Heraeus Pico 17 Centrifuge (Thermo Fisher Scientific Ltd., U.K.), and its protein concentration measured. This step was repeated after 60 days of incubation at room temperature. For samples containing TPP, the protein concentration was determined by measuring the absorbance at 280 nm using a NanoDrop 2000 (Thermo Fisher Scientific Ltd., U.K). For samples containing ATP, the concentration was measured using the Pierce BCA Protein Assay Kit assay (Thermo Fisher Scientific Ltd., U.K.) per manufacturer’s protocol. A stock solution of lysozyme with a known concentration was used to prepare the dilution series of standard samples to obtain the standard protein concentration curve.

### DLS Measurements

DLS measurements were carried out on a Wyatt DynaPro Platereader (Wyatt Technology Corporation, Santa Barbara, CA 93117). The samples were made to the desired protein and excipient concentrations in an Eppendorf tube, centrifuged at 10,000 rpm for 10 min to remove solid precipitate, and finally passed through a 0.02 μm Whatman Anotop syringe filter (Scientific Laboratory Supplies Ltd, Nottingham, U.K.) into a new Eppendorf tube. For each experiment, 25 μL of 0.02 μm filtered samples were loaded into a low volume Corning 384-well microplate (Merck KGaA, Darmstadt, Germany). Each sample was run in triplicates, and the acquisition time was set to 5 s. Cumulant analysis of the intensity auto-correlation function data implemented in the DYNAMICS software (Wyatt Technology Corporation, Santa Barbara, CA 93117) was used to determine the mutual diffusion coefficient *D*, which is related by the Stokes–Einstein relation to an apparent hydrodynamic radius *R*_h_. When measured at a low protein concentration, *R*_h_ is given by
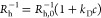
1where *R*_h,0_ is the hydrodynamic radius of the protein and the diffusion interaction parameter *k*_D_ accounts for the effects of hydrodynamic and thermodynamic interactions on the diffusion coefficient. By setting *R*_h,0_ equal to 1.9 nm in eq 1, we obtain apparent values of *k*_D_, which provide a direct indication of the interparticle interactions. The values are expected to be accurate because the polydispersity values determined from cumulant analysis fits were less than 0.1 in all cases and the *R*_h,0_ value for lysozyme was found to be invariant with salt concentration and salt type.^82^

### ζ-Potential Measurements

ζ-Potentials of lysozyme in the presence of varying concentrations of ATP, TPP, and ATP–Mg were acquired on a Zetasizer Nano ZSP (Malvern Instruments Ltd., Malvern, UK) using DTS1070 folded capillary cells (Malvern Instruments Ltd., Malvern, UK). All ζ-potential measurements were made with 1 mg/mL protein concentration at 25 °C. Henry’s function was set equal to 1.5 according to the Smoluchowski approximation. The sample was allowed to equilibrate for 30 s before 20 measurements were collected and averaged. Each sample condition was repeated five times with error bars corresponding to the standard deviation across replicate measurements.

### NMR Experiments

All NMR spectra were acquired at 25 °C on an 800 MHz Bruker Avance III spectrometer equipped with a 5 mm triple resonance TCI cryoprobe and temperature control unit. The spectra were acquired and processed using Bruker Topspin 4.0.8 (Bruker), while the further analysis was done using OriginPro9.1 (OriginLabs) and NMRFAM-Sparky. Samples for NMR were prepared by adding 5% v/v ^2^H_2_O to 500 μL of 10 mg/mL lysozyme in 10 mM Tris, pH 7.0, and transferred to 5 mm NMR tubes (Wilmad). ATP or TPP were then gradually titrated into the NMR sample and mixed prior to data acquisition.

The side-chain chemical shifts were monitored by a natural abundance ^1^H–^13^C HSQC experiment with sensitivity enhancement, gradient coherence selection, and multiplicity editing, which enabled us to easily distinguish between CH_2_ and CH/CH_3_ groups. To obtain the ^1^H–^13^C assignments of lysozyme at pH 7.0, we first transferred assignments from BMRB ID 4562 to the lysozyme spectrum obtained at pH 3.6 by matching peak positions and then gradually titrated the sample to pH 7.2 to follow the peak positions and obtain a reliable assignment at pH 7.0 (Figure S14).

Chemical shift changes in the ^1^H spectrum were calculated using eq 2.
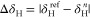
2

δ_H_^ref^ and δ_H_^*n*^ represent the proton chemical shift of the peaks in a lysozyme sample without and with added ATP/TPP, respectively. The chemical shifts in ^1^H–^13^C HSQC were calculated using eq 3., where Δδ_H_ and Δδ_C_ represent the chemical shift changes in proton and carbon dimensions, respectively.
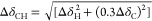
3

Threshold of significance was calculated using the Schumann method.^48^ CSP were fitted to eq 4 describing 1:1 binding, except for W62, which was fitted to a 2:1 binding model using OriginPro9.1 software (OriginLabs).
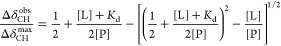
4Δδ_CH_^obs^ is the observed chemical shift from the free state, Δδ_CH_^max^ maximal chemical shift upon complex saturation, [P] total soluble protein concentration, [L] total ATP concentration, and *K*_d_ dissociation constant.

### X-ray Crystallography

Crystal screening of lysozyme was performed by sitting-drop vapor diffusion by mixing 200 nL of protein at 20 mg/mL in buffer (10 mM Tris–HCl pH 7.0) with an equal volume of reservoir solution and incubating the plates at 21 °C. The reservoir solutions contained 10 mM Tris–HCl at pH 7.0, and the ATP/TPP concentration was varied between 0 and 80 mM. Crystals of the lysozyme–ATP complex were obtained in various conditions, with the best quality data collected on crystals from a condition containing 10 mM Tris–HCl pH 7.0, 80 mM Na_2_ATP, and 20 mg/mL protein at 294 K. Lysozyme–TPP complex crystals were obtained in a single condition containing 10 mM Tris–HCl pH 7.0 and 40 mM Na_5_TPP. Crystals were cryoprotected in perfluoropolyether cryo oil (PFO) prior to flash cooling in liquid nitrogen. Data were subsequently collected at the io3 beamline at Diamond Light Source and scaled and merged with Xia2. The resolution cut-off was determined based upon the CC 1/2 and paired refinement in PDB-Redo. Preliminary phases were obtained by molecular replacement in Phaser using a search model derived from Protein Data Bank (PDB) entry 2LZT. Crystals of the lysozyme–TPP complex diffracted at a resolution above 4 Å, and further structure determination was not attempted. For the lysozyme–ATP complex, iterative cycles of model building in Coot and refinement in Phenix.refine were used to generate the completed model of the lysozyme–ATP complex structure. Validation with MolProbity was integrated into the iterative rebuild and refinement process. The crystal structure of the lysozyme–ATP complex was solved at 1.27 Å resolution with 0.87, 0.98, and 0.79 occupancy of the ATP binding sites. Complete data collection and refinement statistics are presented in Table 1. The coordinates of the lysozyme–ATP complex structure were deposited at PDB (ID 8AAZ).

## Supporting Information Available

Protein concentration data, photo of lysozyme crystals, details of ATP–ATP interaction in the crystal structure, visualization of dimers in the crystal lattice, ^13^C HSQC CSP data for NMR titration experiment with ATP, visualization of arginine–lysine pairs on the lysozyme surface, ^31^P CSP data, ^1^H NMR spectra of lysozyme at various concentrations of ATP–Mg, ^13^C HSQC CSP data for NMR titration experiment with ATP–Mg, ^1^H NMR spectra of lysozyme in the presence of ATP–Mg and NaCl, visual comparison of ATP–Mg binding sites on the lysozyme surface determined by NMR and MD simulations, NMR spectra of the HSA–ATP complex, ^13^C HSQC spectra of lysozyme at different pHs, details of ATP and TPP binding sites, additional references, and additional experimental procedures (PDF)

## Abbreviations

ATP: adenosine tripolyphosphate
DLS: dynamic light scattering
NMR: nuclear magnetic resonance
TPP: tripolyphosphate
CSP: chemical shift perturbation
