# Supplementary material for: Nonspecific Binding of Adenosine Tripolyphosphate and Tripolyphosphate Modulates the Phase Behavior of Lysozyme
Source: J Am Chem Soc. 2023 Jan 6;145(2):929–43. doi: 10.1021/jacs.2c09615 (PMC9853864; doi:10.1021/jacs.2c09615)
Supplement: Supplementary file 1 — ja2c09615_si_001.pdf [file ja2c09615_si_001.pdf]

Supplementary information for

Nonspecific binding of adenosine tripolyphosphate and tripolyphosphate modulates phase behaviour of lysozyme

Matja Zalar[‡], Jordan Bye[‡†], Robin Curtis[‡*]

[‡]Manchester Institute of Biotechnology, Department of Chemical Engineering, Faculty of Science and Engineering, The University of Manchester, 131 Princess Street, Manchester, M1 7DN, UK

*corresponding author

CONTENTS:

1. **Supplementary Figures ……………………………………………………………...S1**
2. **Supplementary Tables………………………………………………………..…….S10**
3. **Supplementary References……………………………………………………..….S11**
4. **Supplementary Experimental section……………………………………….….. S11**

1. Supplementary Figures

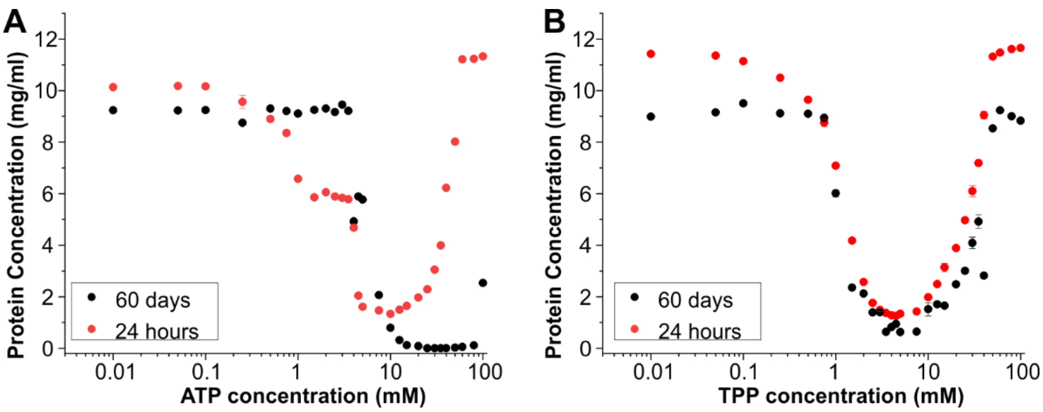

Figure S1: Biophysical characterisation of lysozyme phase transition upon addition of ATP and TPP. Protein concentration measurements immediately after sample preparation (red circles) and after 60 day incubation at room temperature (black circles) of lysozyme solution upon addition of various concentrations of A) ATP and B) TPP

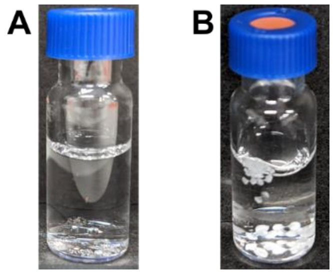

Figure S2: Photo of crystals formed in solution of 10 mg/mL lysozyme in 10 mM Tris buffer, pH 7.0 in the presence of A) 40 mM ATP and B) 40mM TPP. Photos taken 60 days after sample preparation.

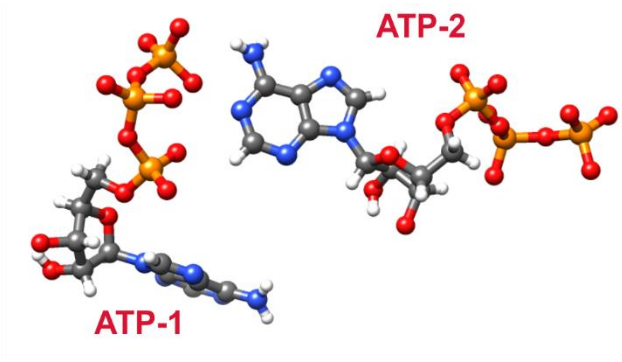

Figure S3: ATP-ATP interaction bound in site I (ATP-1) and site II (ATP-2) on lysozyme as identified by the crystal structure. Protein atoms are omitted for clarity of representation.

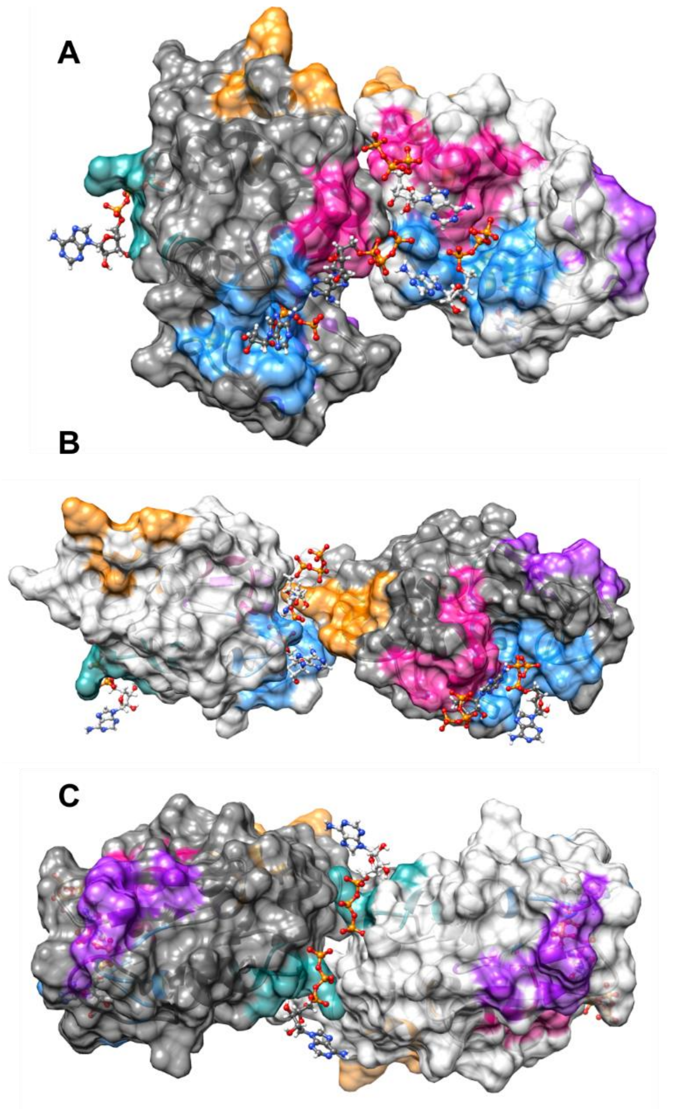

Figure S4: Representation of dimers in the crystal lattice, formed by ATP cross linking interactions. Neighbouring lysozyme molecules, crosslinked by ATP-1 (A), ATP-2 (B) and ATP-3 (C) are shown in surface representation, with ATP molecules shown in stick and ball representation. Lysozyme residues are coloured by the binding sites identified by NMR (Table S1).

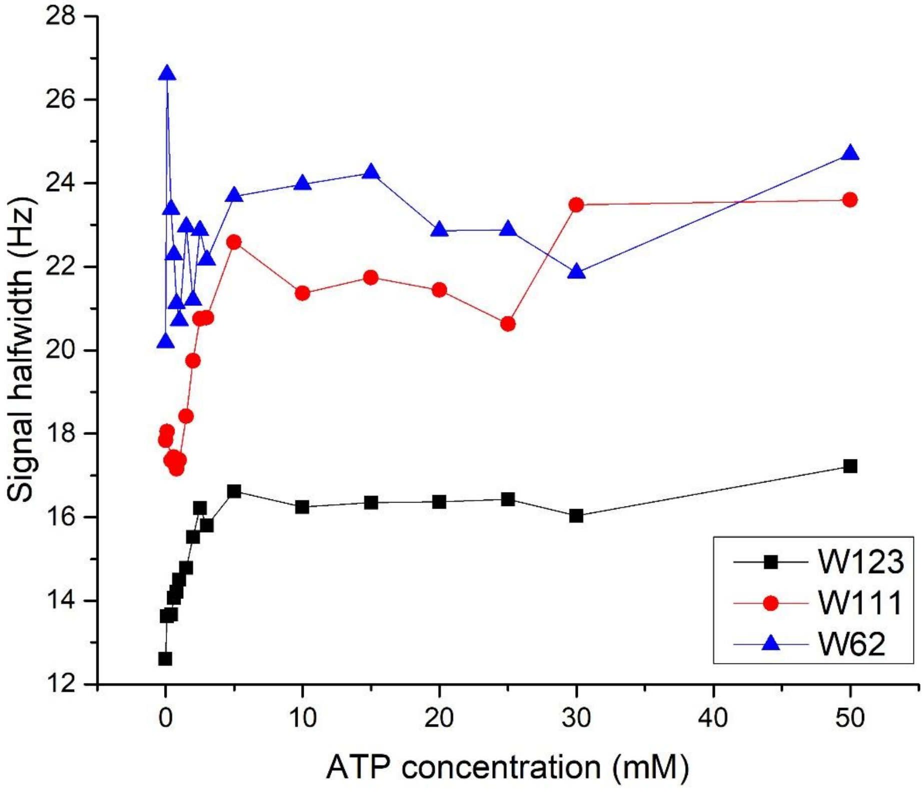

Figure S5: Analysis of the halfwidth of the ^{1}H signals for the binding sites I, II and IV. The increase of the halfwidth corresponds to lysozyme-ATP cluster formation.

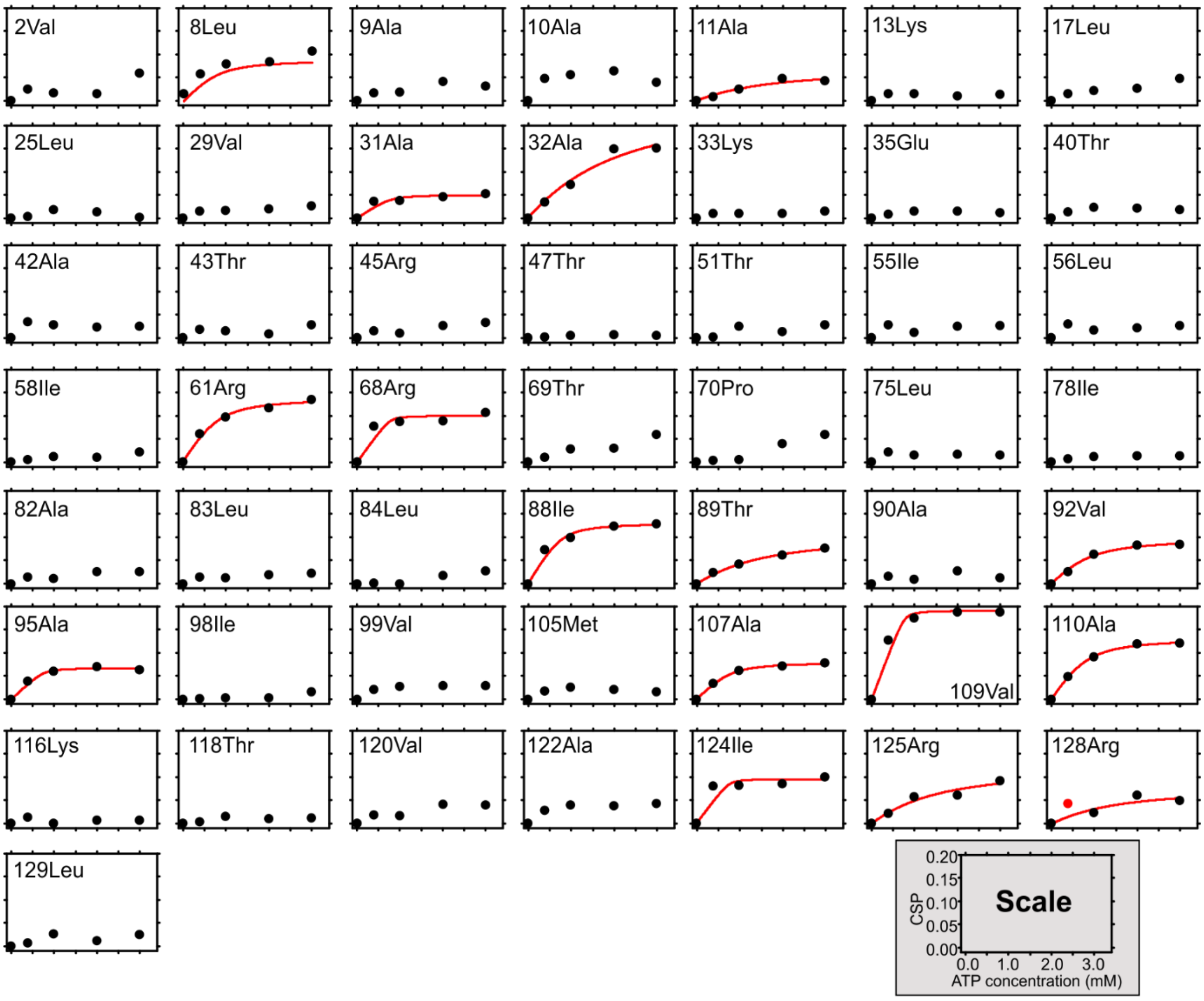

Figure S6: Concentration dependence of chemical shift perturbations (CSP) in the methyl region of ^{13}C HSQC spectra of lysozyme upon gradual titration with ATP. Red lines represent the fits to the quadratic equation for estimation of the binding constants for individual residues.

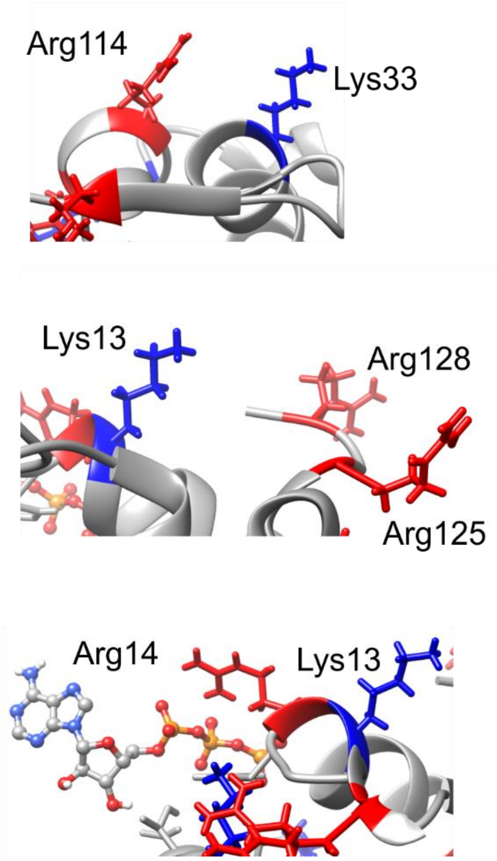

Figure S7: Representation of selected arginine and lysine residues on lysozyme surface. Arginine and lysine residues are shown in red and blue, respectively.

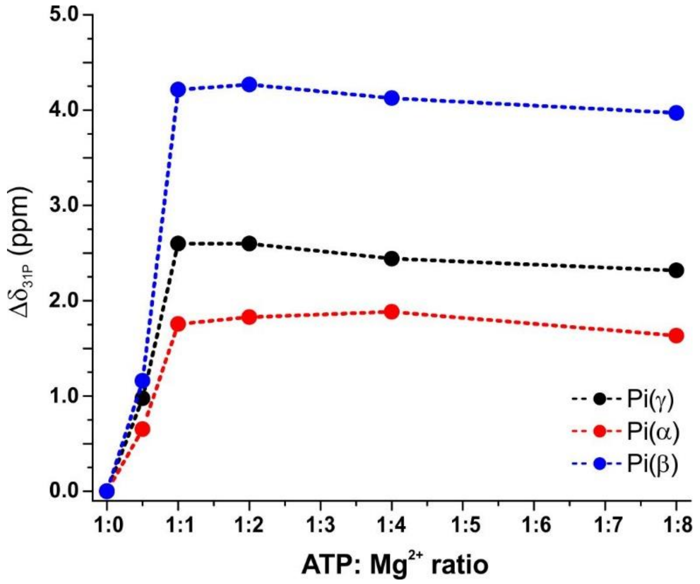

Figure S8: Change of chemical shifts of ATP signals in the ^{31}P NMR spectra $(\Delta\delta_{^{31}P})$ upon gradual addition of $MgCl_2$. ATP-Mg complex is fully formed at equimolar concentration of ATP and Mg^{2+}.

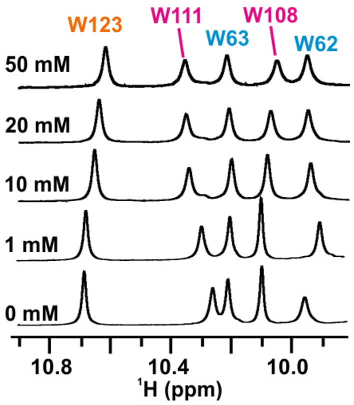

Figure S9: Stack of imino regions of ^{1}H spectra of lysozyme in the presence of various concentration of ATP showing chemical shift perturbations of imino protons. Annotation of residues is shown above signals

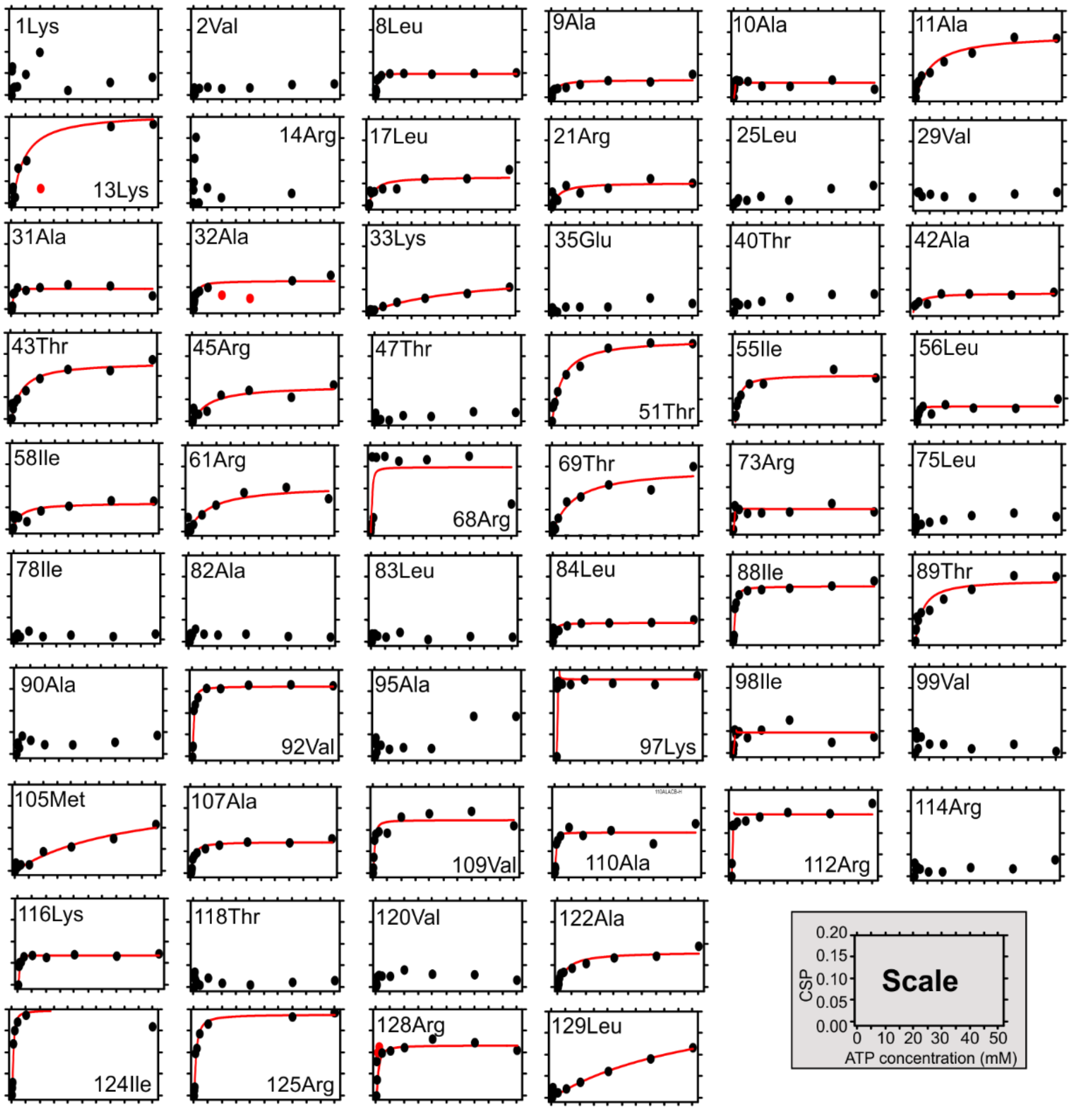

Figure S10: Concentration dependence of chemical shift perturbations (CSP) in the methyl region of ^{13}C HSQC spectra of lysozyme upon gradual titration with ATP-Mg.

Red lines represent the fits to the quadratic equation for estimation of the binding constants for individual residues. Data points colored in red were omitted from analysis.

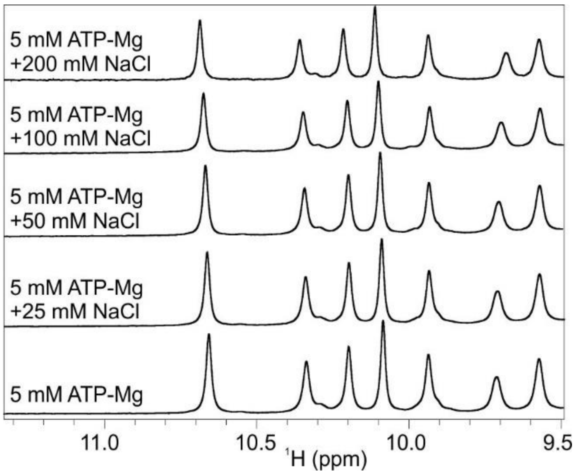

Figure S11: NMR evaluation of ATP-Mg binding to lysozyme in the presence of NaCl
Stack of imino regions of ^{1}H spectra of lysozyme in the presence of 5mM ATP-Mg and increasing concentration of NaCl. The binding of ATP-Mg to lysozyme is not affected by addition of NaCl which screens electrostatic interactions.

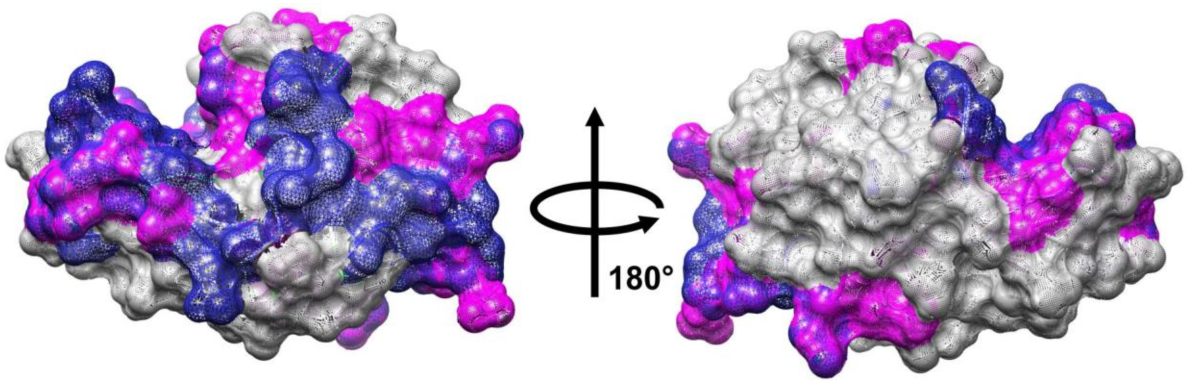

Figure S12: Comparison of ATP-Mg binding sites determined by NMR (pink) and by MD simulations (Blue) as published by Ou *et al*.[1]

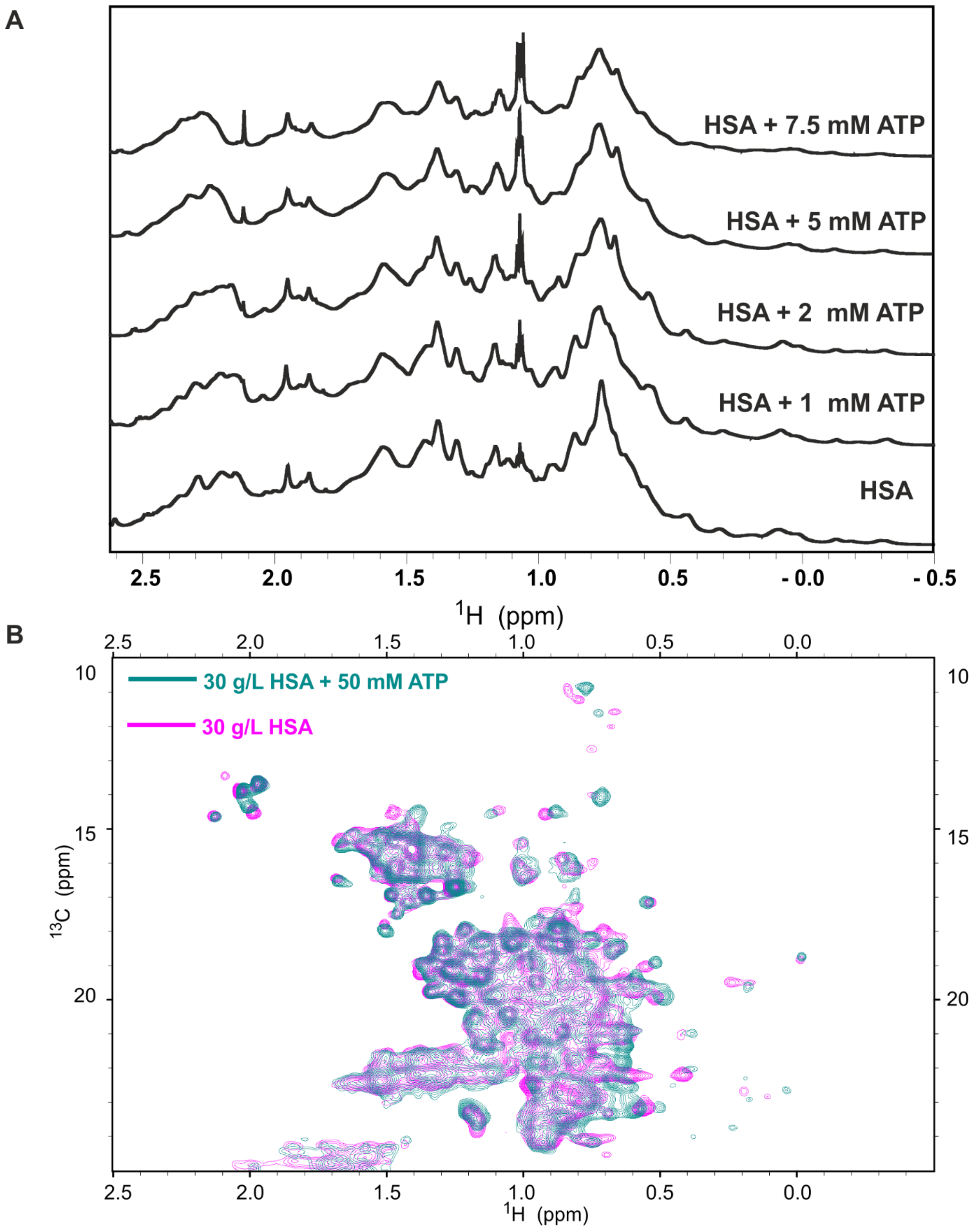

Figure S13: A) Stack of methyl regions of ^{1}H spectra of HSA in the presence of various concentration of ATP showing chemical shift perturbations of the methyl protons. B) Overlay of the methyl region of ^{13}C HSQC spectra of HSA (pink) in the presence of 50 mM ATP (green). Chemical shift perturbations in the methyl and aliphatic regions of HSA NMR spectra upon addition of ATP.

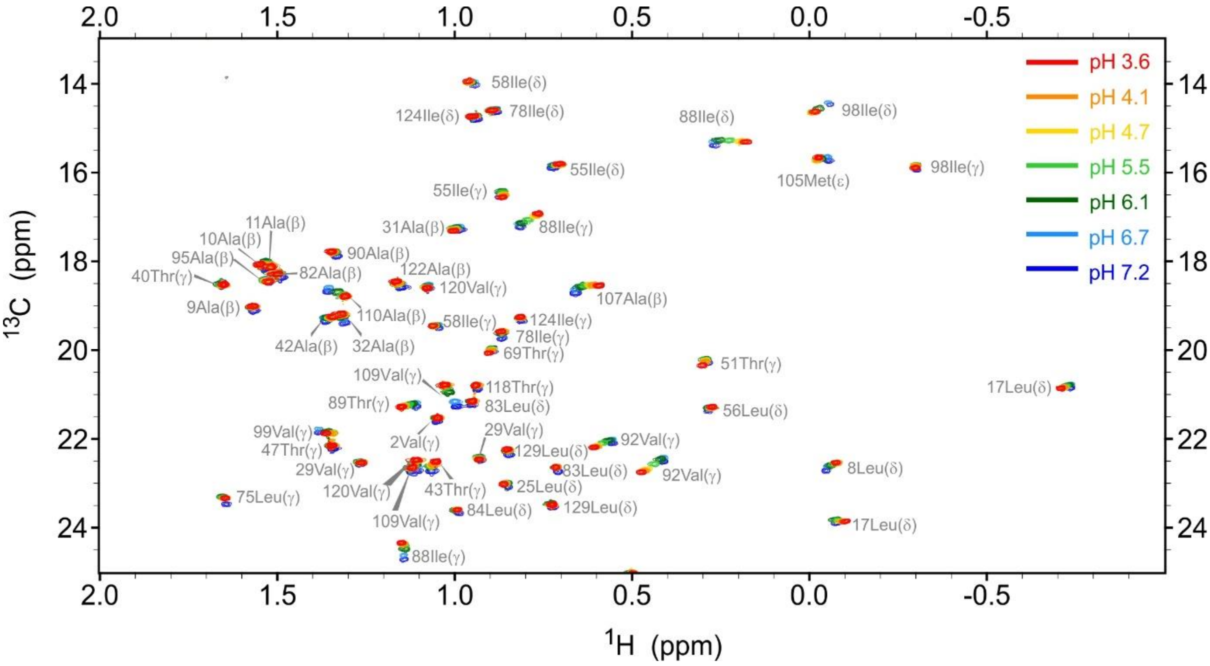

Figure S14: Overlay of ^{13}C HSQC spectra of lysozyme at different pH values. The small chemical shift changes do not alter assignments at neutral pH.

2. Supplementary tables

Table S1: ATP and TPP binding sites as identified by NMR

ATP			
**Binding site**	**Residue Number**	**Colour on Figures**	**Estimated K_d values (mM)[1] *determined from ^{1}H data ** determined from HSQC data**
I	Ile58, Arg61, Trp62, Trp63, Leu75, Ile98	blue	0.80±0.21 mM* 0.04±0.02 mM*
II	Glu35, Met105, Ala107, Trp108, Val109, Ala110	pink	0.94±0.12 mM*
III	Arg14, His15, Ile88, Thr89, Val92	dark cyan	1.043 ± 0.33 **
IV	Val29, Ala122, Trp123, Ile124, Arg125	orange	28.9 ± 3.8 mM *
V	Thr43, Arg45, Thr51, Ile55, Arg68, Thr69	purple	1.1 ± 0.085 mM **
VI	Arg128	red	1.0± 2.2 mM
**TPP**			
**Binding site**	**Residue Number**	**Colour on Figures**	**Estimated K_d values (mM)[1] *determined from ^{1}H data ** determined from HSQC data**
I	Arg61, Trp62, Trp63, Leu75, Ile98	blue	0.94 ± 0.3 mM *
II	Glu35, Ala107, Trp108, Val109, Ala110, Lys116	pink	5.6 ± 1.5 mM *
III	Ile88, Ala90, Val92	dark cyan	/
IV	Trp123, Ile124	orange	0.57 ± 0.11 mM*
V	Thr43, Arg45, Arg68, Leu84	purple	/
VI	Val2, Leu129	red	/
**ATP-Mg**			
**Binding site**	**Residue Number**	**Colour on Figures**	**Estimated K_d values (mM)[1] *determined from ^{1}H data ** determined from HSQC data**
I	Ile58, Arg61, Trp62, Trp63, Leu75, Ile98	blue	1.4.±1.2 mM* 0.004±0.04 mM*
II	Glu35, Met105, Ala107, Trp108, Val109, Ala110, Trp111	pink	1.2±0.2 mM*
III	Arg14, His15, Leu17, Arg21, Ile88, Thr89, Val92	dark cyan	1.6 ± 1**
IV	Val29, Ala122, Trp123, Ile124, Arg125	orange	16.5 ± 1.0 mM *
V	Thr43, Arg45, Thr51, Ile55, Arg68, Thr69	purple	1.0 ± 0.3mM **
VI	Arg128, Leu 129	red	0.33 ± 0.52 mM

$^{1}K_d$ values are reported for single residue within the binding site

3. Supplementary references

1. Ou, X.; Lao, Y.; Xu, J.; Wutthinitikornkit, Y.; Shi, R.; Chen, X.; Li, J., ATP Can Efficiently Stabilize Protein through a Unique Mechanism. *JACS Au* **2021,** *1* (10), 1766-1777.

4. Supplementary experimental section

Human serum albumin sample preparation and NMR experiments

Human serum Albumin (HSA) with a purity of ≥97%, was purchased from Sigma Aldrich (Sigma Aldrich, Gillingham, UK, product number A9511.

Stock solution of HSA was prepared by dissolving the protein in 10 mM Tris buffer, pH 7.0 and then dialysed against 600 mL of the buffer solution for 4 h twice and again overnight. After dialysis, the pH of the protein stock solutions were checked again and adjusted to pH 7.0 (±0.05) if needed, and the protein stock concentration was adjusted to 40 mg/mL. Finally, the HSA stock solution was filtered through a series of 0.22 µm and 0.1 µm hydrophilic nylon membranes (Merck Millipore Ltd., Ireland). Protein concentrations were determined by measuring UV-absorption at 280 nm using NanoDrop 2000 (Thermo Fisher Scientific).

NMR spectra were acquired at 25 °C on 800 MHz Bruker Avance III spectrometer equipped with 5 mm triple resonance TCI cryoprobe and temperature control unit. The spectra were acquired and processed using Bruker Topspin 4.0.8 (Bruker). Samples for NMR were prepared by addition of 5% v/v $^{2}H_2O$ to 500 µL of 30 mg/ml HSA in 10 mM Tris, pH 7.0 and transferred to 5 mm NMR tubes (Wilmad). ATP was then gradually titrated into the NMR sample and mixed prior to data acquisition.
